# Genome-wide chromosomal association of Upf1 is linked to Pol II transcription in *Schizosaccharomyces pombe*

**DOI:** 10.1093/nar/gkab1249

**Published:** 2021-12-20

**Authors:** Sandip De, David M Edwards, Vibha Dwivedi, Jianming Wang, Wazeer Varsally, Hannah L Dixon, Anand K Singh, Precious O Owuamalam, Matthew T Wright, Reece P Summers, Md Nazmul Hossain, Emily M Price, Marcin W Wojewodzic, Francesco Falciani, Nikolas J Hodges, Marco Saponaro, Kayoko Tanaka, Claus M Azzalin, Peter Baumann, Daniel Hebenstreit, Saverio Brogna

**Affiliations:** School of Biosciences and Birmingham Centre of Genome Biology (BCGB), University of Birmingham, UK; Division of Cellular and Gene Therapies, Tumor Vaccines and Biotechnology Branch, Center for Biologics and Evaluation Research, US Food and Drug Administration, Silver Spring, MD, USA; School of Life Sciences, University of Warwick, Coventry, UK; School of Biosciences and Birmingham Centre of Genome Biology (BCGB), University of Birmingham, UK; School of Biosciences and Birmingham Centre of Genome Biology (BCGB), University of Birmingham, UK; School of Biosciences and Birmingham Centre of Genome Biology (BCGB), University of Birmingham, UK; School of Biosciences and Birmingham Centre of Genome Biology (BCGB), University of Birmingham, UK; School of Biosciences and Birmingham Centre of Genome Biology (BCGB), University of Birmingham, UK; Interdisciplinary School of Life Sciences, Banaras Hindu University, Varanasi 221005, India; School of Biosciences and Birmingham Centre of Genome Biology (BCGB), University of Birmingham, UK; School of Biosciences and Birmingham Centre of Genome Biology (BCGB), University of Birmingham, UK; School of Biosciences and Birmingham Centre of Genome Biology (BCGB), University of Birmingham, UK; School of Biosciences and Birmingham Centre of Genome Biology (BCGB), University of Birmingham, UK; Department of Microbial Biotechnology, Faculty of Biotechnology and Genetic Engineering, Sylhet Agricultural University, Sylhet 3100, Bangladesh; School of Biosciences and Birmingham Centre of Genome Biology (BCGB), University of Birmingham, UK; School of Biosciences and Birmingham Centre of Genome Biology (BCGB), University of Birmingham, UK; Department of Environmental Health, Norwegian Institute of Public Health, Oslo, Norway & Department of Research, Cancer Registry of Norway, Oslo University Hospital, Oslo, Norway & Environmental Genomics, School of Biosciences, University of Birmingham, Birmingham, UK; School of Biosciences and Birmingham Centre of Genome Biology (BCGB), University of Birmingham, UK; School of Biosciences and Birmingham Centre of Genome Biology (BCGB), University of Birmingham, UK; Institute of Cancer and Genomic Sciences, University of Birmingham, UK; Department of Molecular and Cell Biology, University of Leicester, UK; Instituto de Medicina Molecular João Lobo Antunes (iMM), Faculdade de Medicina da Universidade de Lisboa, Lisbon, Portugal; Johannes Gutenberg University, Mainz, Germany; School of Life Sciences, University of Warwick, Coventry, UK; School of Biosciences and Birmingham Centre of Genome Biology (BCGB), University of Birmingham, UK

## Abstract

Although the RNA helicase Upf1 has hitherto been examined mostly in relation to its cytoplasmic role in nonsense mediated mRNA decay (NMD), here we report high-throughput ChIP data indicating genome-wide association of Upf1 with active genes in *Schizosaccharomyces pombe*. This association is RNase sensitive, correlates with Pol II transcription and mRNA expression levels. Changes in Pol II occupancy were detected in a Upf1 deficient (*upf1Δ*) strain, prevalently at genes showing a high Upf1 relative to Pol II association in wild-type. Additionally, an increased Ser2 Pol II signal was detected at all highly transcribed genes examined by ChIP-qPCR. Furthermore, *upf1Δ* cells are hypersensitive to the transcription elongation inhibitor 6-azauracil. A significant proportion of the genes associated with Upf1 in wild-type conditions are also mis-regulated in *upf1Δ*. These data envisage that by operating on the nascent transcript, Upf1 might influence Pol II phosphorylation and transcription.

## INTRODUCTION

Upf1 is a conserved protein of eukaryotes that has so far been primarily studied for its key role in nonsense-mediated mRNA decay (NMD). NMD is a translation-coupled cytoplasmic mechanism believed to recognise and rapidly destroy mRNAs carrying a premature termination codon or other features that place a stop codon in abnormal sequence contexts (1–8). However, the precise role of Upf1 in NMD as well as NMD significance and mechanisms remain unclear (9,10).

Upf1 belongs to the 1B superfamily (SF1B) of helicases that are involved in a diverse range of cellular activities in all domains of life. These are characterised by conserved sequence motifs and the ability to translocate in a 5′ to 3′ direction on both RNA and DNA (11,12). Specifically, there is evidence that Upf1 uses ATP hydrolysis to translocate on RNA and to displace RNA-bound proteins (13–17).

In yeast, as in other organisms, Upf1 is typically most abundant in the cytoplasm. For this reason, it is assumed that Upf1 operates on mRNAs only after their nuclear export. However, there is evidence to show that Upf1 traffics in and out of the nucleus in mammalian cells (18,19). It was initially proposed that within the nucleus Upf1 plays a direct role in DNA replication, telomere maintenance and DNA repair (20). However, the effects of Upf1 depletion on DNA replication and cell division might be an indirect consequence of changes in the expression of genes involved in these processes (21,22). There is circumstantial evidence that Upf1 might instead play a direct role in RNA-based processes of gene expression within the nucleus (22).

The putative association of Upf1 with chromatin was examined by chromatin immunoprecipitation (ChIP) in *Schizosaccharomyces pombe* with the aim to understand what roles Upf1 may have in the nucleus. The data demonstrate Upf1 binding to chromatin and indicate that this occurs primarily at active genes. This association is genome-wide and positively correlates with RNA Pol II loading and mRNA expression. Notably, this interaction is RNase sensitive and Upf1 does not co-purify with Pol II directly. These data therefore indicate that Upf1 binds the nascent transcript. Upf1 depleted cells show abnormalities in Pol II loading, CTD phosphorylation and are also hypersensitive to the 6-azauracil, a drug that can affect transcription elongation. Genes that are associated with Upf1 in wild-type conditions are more likely to be mis-regulated in *upf1*Δ cells. Cumulatively these findings predict that Upf1, by operating on nascent mRNA Pol II genes, can regulate their transcription.

## MATERIALS AND METHODS

### Yeast strains and methods

The complete list of *S. pombe* strains used in this study is shown in Supplementary Table S1. Fission yeast transformation was carried out as described earlier (23). The target proteins were HA and Flag-tagged by homologous recombination using a PCR-amplified fragment containing the kanMX6 or hphMX6 cassette flanked by targeting sequences (24). All PCR primers used for tagging target genes are listed in Supplementary Table S2.

### Western blotting

Protein extraction from *S. pombe* cells was done as described before (25). Membranes were probed with the required primary antibodies: rabbit anti-Flag (F7425, Sigma Aldrich), rat anti-HA (11867423001, Sigma Aldrich), mouse anti-alpha-tubulin (AB_477579, Sigma- Aldrich, 1:2500), rat anti-Ser2 Pol II (AB_11212363, Merck Millipore, 1:5000). Respective secondary antibodies, IRdye 800 and/or 680 were used to detect the signal using an Odyssey infra-red imaging system (LI-COR Biosciences).

### ChIP

Freshly harvested cells from exponentially growing cultures (OD_600_ = 0.5) were fixed for 5 min at room temperature with 1% formaldehyde (Sigma Aldrich) followed by 10 min incubation with a further addition of glycine to stop the cross-linking following published yeast ChIP protocols (26). The cell pellet was collected and washed twice with ice-chilled 1X PBS with spinning at 5000 rpm for 3 min each. The pellet was resuspended in ice-cold FA lysis buffer [HEPES–KOH-100 mM (pH 7.5), NaCl 300 mM, EDTA 2mM, Triton X-100 2%, Na-Deoxycholate 0.2%] containing 1X protease inhibitor (EDTA-free protease inhibitors cocktail tablet, Roche). Cells were pelleted at 6000 rpm for 2 min at 4°C and the pellet was resuspended in FA lysis buffer and Zirconia beads (0.7 mm diameter, Biospec). Cells were broken using a cell homogenizer (Bertin Instruments, Precellys 24, 10 cycles: 30 s at 5500 rpm and 2 min in ice). The bottom of each screw cap tube was pierced three times with a red-hot 25 G needle and each tube was immediately transferred to the barrel of a syringe fitted in a 15 ml falcon tube. The lysate was collected at 1000 rpm for 1 min at 4°C. To increase sonication efficiency and prevent proteases, 20 μl of 10% SDS and 20 μl of 100 mM PMSF were added to the mixture. Samples were sonicated for 15 cycles using a Bioruptor (Diagenode), to generate ∼500 bp average fragment size. Immunoprecipitation was done by adding Dynabeads (Thermofisher) and incubated overnight at 4°C on a rotor. The supernatant was removed and beads were washed for 5 min at room temperature on a rotor using buffers as mentioned: Wash Buffer I [HEPES–KOH 50 mM (pH 7.5), NaCl 150 mM, EDTA 1 mM (pH 8.0), Triton X-100 1%, sodium deoxycholate 0.1%, SDS 0.1%) 2 times; Wash Buffer II [HEPES–KOH 50 mM (pH 7.5), NaCl 500 mM, EDTA 1 mM (pH 8.0), Triton X-100 1%, Na-deoxycholate 0.1%, SDS 0.1%]—2 times; Wash Buffer III [Tris–HCl 10 mM (pH 8.0), EDTA 1 mM (pH 8.0), LiCl 0.25 mM, IGEPAL CA630 0.5%, Na-deoxycholate- 1%]—2 times and TE [Tris–HCl- 10 mM (pH 8.0), EDTA 1 mM (pH 8.0)]—2 times. After the final wash, beads were resuspended in 100 μl Elution Buffer (EB) [Tris–HCl 50 mM (pH 7.5), EDTA 10 mM, SDS 1%] and incubated for 10 min at 65°C and occasionally vortexed. The supernatant (elution) was recovered and transferred to a fresh 1.5 ml DNA low bind tube. To the input, 150 μl EB was added and incubated at 65°C overnight to allow de-crosslinking. The IP sample was de-crosslinked in parallel using the same condition. To remove proteins from the DNA, 5 μl Proteinase K (20 mg/ml) was added and samples were incubated at 50°C for 2 h. DNA was then extracted using the Monarch PCR purification kit, as previously described (27).

RNase ChIP, radioactive PCR and ChIP-chip were carried out as previously described (23). qPCR quantification of DNA samples was carried out using the SensiFAST SYBR Hi-ROX Kit (Bioline, BIO-92005) in 96-well plates using a ABI PRISM 7000 system (Applied Biosystems); primer sequences are listed in Supplementary Table S2. For ChIP-seq, all ChIP-DNA libraries were produced using the NEBNext Ultra II DNA Library Prep Kit (NEB, E7645L) and NEBNext Multiplex Oligos for Illumina (NEB, E7600S), using provided protocols with 10 ng of fragmented ChIP DNA. Pipetting was done with a Biomek FxP robotic work station (Beckman Coulter, A31842). Constructed libraries were assessed for quality using the TapeStation 2200 (Agilent, G2964AA) with High Sensitivity D1000 DNA ScreenTape (Agilent, 5067–5584).

### Analysis of ChIP-chip data

We used the Model-based Analysis of Tiling Arrays (MAT) software to analyse the Affymetrix hybridization data (28). ChIP input DNA sample was used as the control and was compared against Upf1 (asynchronous and S-phase) and Pol II samples. A *P*-value cut off of 10^–4^ or 10^–3^ was used, whereas the remaining MAT parameters remained as default. Results produced by the MAT software were visualised in Affymetrix's Integrated Genome Browser (IGB) (29). When 50% or more of a genomic region was significantly bound by Upf1 and Pol II, we called it an enriched gene/genomic region. Enrichment scores were assigned to genomic features using the *S. pombe* genome coordinates (ftp://ftp.sanger.ac.uk/pub/yeast/pombe/GFF). The average enrichment was calculated between the start and end coordinates of enriched genomic regions, thereby giving each enriched region a score based on fold enrichment. Identification of significantly bound genomic features and enrichment score calculation was done using the statistical computing language R (http://www.R-project.org/). Functional annotation of the enriched regions was done using DAVID (30).

### Pol II and Upf1 purification

Exponentially growing cultures (OD_600_ 0.5) of healthy *S. pombe* cells were pelleted down for 10 min at 5000 rpm at 4°C (Rotor: F10-6 × 500, FiberLite Beckman J2-MC). The pellet was washed with buffer 1 (HEPES 20 mM, KAc 110 mM) and resuspended in lysis buffer [HEPES 20 mM, KAc 110 mM, Triton X-100 0.5%, Tween 20 0.1%, MnCl_2_ 10 mM, PMSF 1 mM, protease inhibitor 1X, PhosSTOP-1X (Roche), RNase inhibitor 50 U/ml, RVC 10 mM]. Small droplets of the lysate were made in liquid nitrogen and immediately kept at −80°C, they were typically processed the day after. Cells were subjected to grinding with SPEX SamplePrep 6775 freezer mill (grind cycle: precool- 2 min, 1 cycle of 1 min cooling & 2 min grinding, impact rate 14). An equal volume of lysis buffer and 110 U/ml of DNase (DNase I recombinant, RNase-free solution, Roche) was added to the ground cell lysate followed by incubation for 1 h at 4°C. The sample was centrifuged at 16 000g for 10 min and the supernatant was transferred to a fresh tube. The supernatant (input) was incubated with 5 μg of anti-Flag antibody-coated Dynabeads for 1 h at 4°C on a rotator. After incubation, Dynabeads were washed 6 times for 10 mins each with wash buffer (HEPES 20 mM, KAc 110 mM, Triton X-100 0.5%, Tween 20 0.1%, RNase inhibitor 50 U/ml, MgCl_2_ 4 mM). Beads were incubated with elution buffer (lysis buffer, MgCl_2_ 4 mM, Flag-peptide 2 mg/ml) for 30 min at 4°C on a rotator. The elution fraction was collected by separating the beads using a magnet.

### ChIP-chip data processing and correlation analysis

For the ChIP-chip data correlation analysis, the raw ChIP-chip probe intensities were processed according to the data preparation and expression value calculation sections of the Affymetrix statistical algorithms description document (http://tools.thermofisher.com/content/sfs/brochures/sadd_whitepaper.pdf) in order to obtain a signal value for each gene for each array. The published Ser5 Pol II ChIP-chip datasets were downloaded from https://www.ebi.ac.uk/arrayexpress/experiments/E-MTAB-18/ (31). The signal values in the two pairs of input control datasets (one pair for the Upf1 IPs and one for the Pol II IPs) underwent some additional processing. For each pair of control datasets, the two sets of signal values were scaled to each other so that they had the same median, before taking the mean to obtain a single signal value for each gene for each pair (one for Upf1 controls and one for Pol II controls). The pair of ChIP-chip datasets with Pol II IP were also combined into one in the same manner described for the control dataset pairs. One of the two datasets from each of the asynchronous and S-phase Upf1 pairs of IP datasets were found to be of low quality. Therefore, only the higher quality dataset from each pair was used. The signal value of each gene in each IP dataset was then normalised by dividing by the signal value of that gene in the appropriate control dataset. These normalised signal values were used for the correlation analysis.

### ChIP-chip metagene analysis

The individual probe signal values from the CEL file corresponding to each of the previously discussed arrays were extracted and associated with their probe sequence and the gene they map to, using the Sp20b_M_v04 chip description file (https://www.ncbi.nlm.nih.gov/geo/query/acc.cgi?acc=GPL10187). The positions of each probe within their corresponding gene were obtained by matching the probe sequence to that of the gene's fasta sequence including coding sequence, introns and UTRs (https://www.pombase.org/downloads/genome-datasets). The probe position was calculated as the percentage through the gene of the 5′-most base's mapping, from the TSS (0%) up to 24 bp upstream of the TES (100%) since the probes are 25 bp in length. We constructed metagenes based on two Upf1 IP arrays, one with asynchronous and one with S-phase synched cells and a pair of corresponding control arrays, as well as a pair of Ser5 Pol II IP arrays along with their pair of corresponding control arrays. Firstly, for each probe in each array, we calculated the ratio of perfect match (PM) and mismatch (MM) probe signal values. Then for the pairs of Upf1 and Pol II control arrays we calculated the mean of the PM / MM signal ratio from each probe to obtain Upf1 and Pol II control mean probe signals. The asynchronous and S-phase Upf1 probe signals were then divided by the Upf1 control mean probe signals to obtain their normalised values, which were used in for plots. For the pair of Pol II IP arrays, each one was normalised by dividing by the Pol II control mean probe signals, and the mean of these normalised values was taken for each probe to obtain the mean normalised Pol II signal. The metagene plots themselves show the average of the values calculated from probes mapping to each 0.1% block of gene bodies.

### ChIP-seq

Exponential cultures of *S. pombe* growing at 30ºC in 400 ml YES with an OD_600_ 0.8 were fixed by 1% formaldehyde at room temperature for 5 min followed by the addition of glycine to 0.125 M final concentration. The fixed cells were washed with ice-cold PBS, resuspended in cell lysis buffer [HEPES 50 mM (pH 7.6), EDTA 1 mM (pH 8.0), NaCl 150 mM, Triton X-100 1%, Na-Doc- 0.1% and 1X protease inhibitor (EDTA-free protease inhibitors cocktail tablet, Roche], and lysed by acid washed glass beads. Chromatin extracted from cell lysates was fragmented by 8 sonicating cycles of 5 min with 30 s ON/30s OFF at HIGH setting (Bioruptor^®^ Plus). Immunoprecipitations were performed using Protein G Dynabeads (Life Technologies) coated with 10 μg monoclonal anti-Flag M2 antibody (Sigma). Both IP and Input DNA were purified using MinElute PCR Purification Kit (QIAGEN). 10 ng of DNA was used for DNA library construction with the NEBNext Ultra II DNA Library Prep Kit (New England Biolab E7645L), indexed using NEBnext Multiplex Oligos for Illumina Dual Index Primers (New England Biolabs E7600S) and sequenced simultaneously using a Illumina HiSeq4000 System.

### ChIP-seq data analysis

The sequence reads in the FASTQ files were trimmed using Trimmomatic to remove low quality reads (32). The SE (single end) setting was used, with sliding window and minimum read length set to 4:22 and 32, respectively, while all other parameters were set to default. The trimmed FASTQ files were then converted to SAM files by aligning the reads to the EF2 *S. pombe* genome build (Ensembl), which was downloaded from (https://emea.support.illumina.com/sequencing/sequencing_software/igenome.html?langsel=/gb/). The alignment was carried out using Bowtie2 (33), which was set to automatically filter out unaligned reads using ‘–no-unal’ option. The single-base resolution genome-wide coverage depth for each file was obtained by finding the number of reads mapping to each base position and dividing by the total number of aligned reads for that file to normalise for sequencing depth. The coverage value for each base position in each IP file was then normalised by dividing by the value for that base position in an input control file. The normalised base-wise coverage values for each of the two pairs of Pol II (Rbp3-Flag) ChIP samples (in wild-type and *upf1*Δ cells) were averaged to obtain one set of Pol II coverage values for each strain.

### Gene expression levels quantification

Two replicate *S. pombe* RNA-seq datasets were downloaded from the Gene Expression Omnibus, with sample IDs GSM2803075 and GSM2803077 from the series with ID GSE104546 (https://www.ncbi.nlm.nih.gov/geo/query/acc.cgi?acc=GSE104546), previously described (34). A single mean FPKM value was obtained for each gene by averaging the FPKM value for that gene from the two replicate datasets. The values were transformed via log (FPKM + 1), which was used for plotting and correlations.

### Identification of differentially expressed genes

Previously published whole-genome microarray RNA expression data of *upf1Δ* and wild-type strains data was used (35). Differentially expressed genes were identified from these datasets using significance analysis of microarrays (SAM) at time point 0 between wild-type and *upf1Δ* using a 1% FDR (36). The overlap between differentially expressed genes and Upf1 associated genes was calculated by random sampling, as following: (i) sampling of 420 genes (corresponding to the number of enriched genes at the more stringent *P*-value threshold of 10^–4^ of the MAT software) from 7054 total annotated genes in the version of the genome analysed; (ii) sampling 543 genes from 5280, as previously tested (35); (iii) calculating the overlap between 420 and 543 randomly selected gene sets; (iv) create an overlap distribution by repeating steps 1–3 1000 times and (v) calculate the *P*-value from where the true overlap value (47 genes) falls in the distribution. *P*-values were also calculated using a Fisher's exact hypergeometric test approach as described in https://rdrr.io/bioc/GeneOverlap/man/GeneOverlap.html.

### RT-qPCR and RNA stability quantification

Total RNA was extracted using the hot acid-phenol method (37). Extracted RNA was first subjected to DNase I (1 unit) treatment (Thermo Scientific) at 37°C for 30 mins, followed by incubation with 50 mM EDTA at 65°C for 10 mins. First-strand cDNA was synthesized using Fast Gene Scriptase II cDNA synthesis kit (Nippon Genetics) from 50 ng of total RNA according to the manufacturer's instructions. Real-time PCR quantification was performed using an ABI PRISM™ 7000 Sequence Detection System (Applied Biosystems) according to the manufacturer's instructions. PCR reactions were performed in 96 well plates using qPCRBIO SyGreen Blue Mix Hi-ROX (PCR Biosystems). The 2-ΔCT method was used to calculate the relative levels of expression of the target transcripts and normalised to Rpl32 mRNA or 18S rRNA. To inhibit transcription, the cells were cultured to OD_600_ (∼0.7) and treated with 150 μg/ml 1,10-phenanthroline (Sigma). Cultures were removed at different time points (0, 2, 10, 20, 40 and 60 minutes) after addition of the drug, and immediately transferred to falcon tubes containing 40 ml of ice-cold water prior to RNA extraction.

## RESULTS

### Upf1 associates with protein coding genes genome-wide

**Figure 1.**
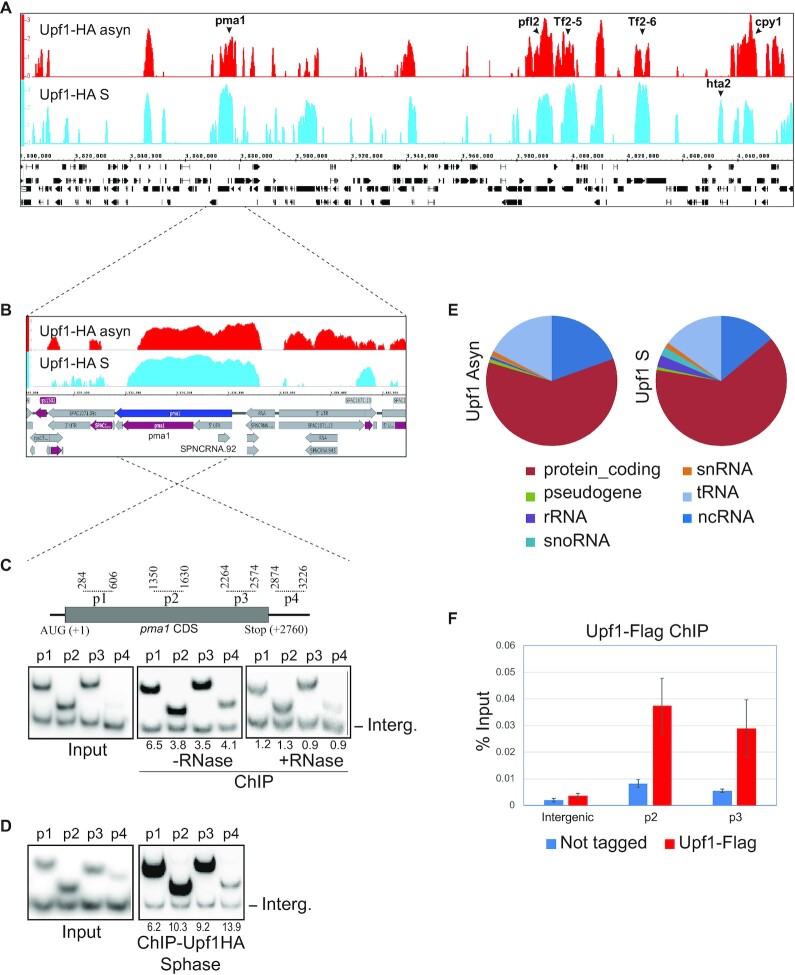
Genome-wide association of Upf1 with protein coding genes. **(A**) IGB visualisation of Upf1-HA ChIP-chip enrichment in asynchronous culture (top track, shown in red) and in S-phase culture (bottom track, shown in sky blue) at a representative chromosomal region (270 kb) of *S. pombe* indicating enrichment of several specific genes, those discussed in the main text are labelled. Genes and genomic features are shown in black below. (**B**) Zoomed-in view of Upf1 enrichment over the entire *pma1* gene (highlighted in dark blue) - 5′UTR (in grey), CDS (in purple) and 3′UTR (in grey) of *pma1* are shown in the bottom row schematic. (**C**) Top panel- diagram of the *pma1* gene (cDNA region in grey) and the positions of amplicons used for the PCR-ChIP assay are indicated by the dotted lines above (numbers correspond to the position of the primers relative to start codon). Bottom panel- polyacrylamide gels showing radiolabelled PCR products produced by the *pma1*-specific primer pairs (top bands) and by the intergenic region specific primer (bottom bands, labelled Int.); using input DNA before ChIP (left panel) and using ChIP-enriched DNA from asynchronous culture without (middle panel) and with RNase pre-treatment of the chromatin (right panel). The relative enrichment of *pma1* DNA relative to intergenic sequence is expressed as a ratio of the intensity of the same fragments produced with the input DNA. (**D**) PCR analysis as in C using input DNA before ChIP (left panel) and using ChIP-enriched DNA from S-phase culture without the RNase treatment (right panel). (**E**) Pie-charts showing the proportion of different classes of gene associated to Upf1 in asynchronous and S-phase culture of *S. pombe*. (**F**) ChIP-qPCR of Upf1-Flag enrichment over *pma1* in three independent asynchronous cultures.

It has been reported that the nuclear level of Upf1 increases upon incubation with leptomycin-B (LMB) in *S. pombe* (Orfeome localization data on Pombase). LMB inhibits the CRM1-dependent protein and some pathways of RNA export, yet most mRNAs are exported by CRM1-independent mechanisms in yeast and human (38,39). Therefore, it is probable that Upf1 is also shuttling between the nucleus and cytoplasm in *S. pombe*. To explore what role it might play in the nucleus, we examined whether Upf1 is associated with individual genes by ChIP. Endogenous *upf1* was tagged with the hemagglutinin (HA) epitope by homologous recombination and ChIP was performed using an HA antibody. This allowed for genome-wide enrichment profiles which were determined by hybridisation of the immunoprecipitated DNA to genomic tiling chip arrays (Affymetrix, see Materials and Methods). We examined both asynchronous and S phase synchronised cell cultures, in duplicate experiments. Significantly enriched regions were identified using the Model-based Analysis of Tiling Arrays (MAT) software (Material and Methods). A total of 594 and 696 genes that are significantly enriched by Upf1 ChIP in 50% or more of their sequence were identified in asynchronous and S phase cultures, respectively (Figure 1 shows the enrichment profiles over a representative region of chromosome 1; Supplementary Table S3 gives the lists of the enriched genes in the two datasets). The Upf1 enrichment profiles are similar between the two samples, however there are several genes enriched more in the S phase sample, such as histone H2A beta (hta2, bold in Figure 1A). Most of the enrichment regions correspond to protein coding genes, which is the class of genes we have investigated in further detail here, yet several non-coding RNA and tRNA genes also appear to be enriched (Figure 1E).

The ChIP-chip data also showed enrichment of repetitive sequences such as centromere and telomere regions, as well as Tf2 retrotransposons (Tf2-5 and Tf2-6 are indicated within the region shown in Figure 1A, and Tf2-9 is shown in Supplementary Figure S1A). The seemingly high ChIP-chip enrichment of some of these repetitive regions may be a technical artefact of the hybridization due to their high sequence similarity. However, Tf2 enrichment was similarly detected in additional ChIP-qPCR experiments using a strain expressing Flag-tagged-Upf1, and was RNase-sensitive (Supplementary Figure S1B). ChIP enrichment of some tRNA genes was also confirmed by qPCR in independent experiments for three arbitrary selected tRNA genes, which is similarly RNase-sensitive (Supplementary Figure S1C).

The ChIP association of Upf1 with protein coding genes was further confirmed by PCR at *pma1* and *act1*, two highly transcribed genes that showed high enrichment in the ChIP-chip datasets. Multiple regions of these genes were examined by radioactive PCR and all showed Upf1 enrichment (Figure 1C-D and Supplementary Figure S2A-2B show *pma1* and *act1* respectively). The association was sensitive to RNase treatment of the chromatin (Figure 1C). This binding of Upf1 with *pma1* was also confirmed by ChIP-qPCR in several later experiments using the Flag-tagged *upf1* strain (Figure 1F and Supplementary Figure S2C). The association with several other active genes was also confirmed by ChIP-qPCR (described in the section below).

### Upf1 chromatin association correlates with Pol II transcription and RNA levels

**Figure 2.**
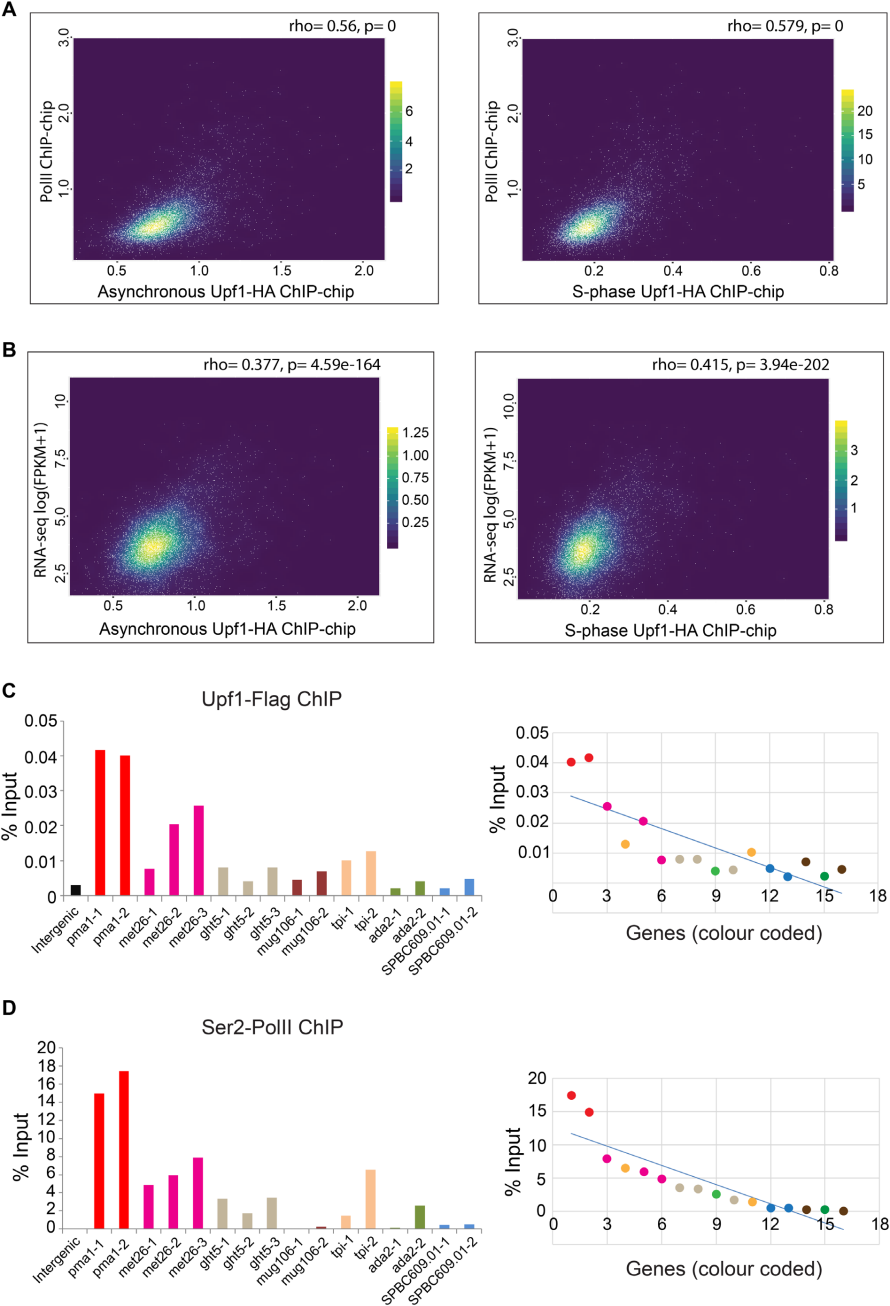
Upf1 association to chromatin positively correlates with Pol II loading and gene expression genome-wide. (**A**) Scatter plots showing genome-wide Upf1(x-axis) versus Pol II (y-axis) ChIP-chip signals in asynchronous (left) and S-phase cultures (right). (**B**) Scatter plots showing genome-wide Upf1 ChIP-chip signals (x-axis) versus RNA-seq signal (y-axis, log(FPKM + 1) in asynchronous and S-phase cultures. rho values indicate correlations analysed with Spearman's rank correlation coefficient, with associated *P*-values (*P* = 0 means *P* < 1e-325). (**C**) Left panel: Upf1-Flag qPCR-ChIP signal on specific regions of seven selected genes and 1 intergenic control (−1, −2 and −3 refer to separate amplicons of the named gene). Genes were selected based on having different levels of Upf1 ChIP-chip association. Right panel- Upf1-Flag ChIP fold enrichment values at specific gene regions plotted according to the Ser2 Pol II ChIP signal, as described in the main text. The primer pairs for each gene are coded with same colour. (**D**) Left panel- Ser2 Pol II ChIP signal on the seven selected genes and the intergenic control. Right panel: the enrichment values for Ser2 Pol II ChIP with each primer pair ordered from high to low values (from left to right). The set of primer pairs for each gene are coded with same colour.

Upf1 ChIP-chip signals were compared with that of Pol II, similarly calculated from a previously published Ser5 Pol II ChIP-chip dataset (Material and Methods). Unlike in other organisms, Ser5 Pol II is not restricted to promoter-proximal regions in *S. pombe*, it is instead loaded throughout the coding region of active genes (31,40). There is a clear correlation between the Upf1 and Pol II ChIP signals at active genes in both the asynchronous and S-phase samples (Spearman's rank correlation test of 0.56 and 0.58 respectively, Figure 2A).

Next, we compared Upf1 enrichment values to gene expression by comparing the Upf1 ChIP-chip signal values with RNA-seq FPKM values for all genes (Material and Methods). Again, a positive correlation is observed between the Upf1 enrichment values from both asynchronous and S phase samples and the RNA-seq FPKM values (based on the top 90% expressed genes according to the RNA-seq data), with a Spearman's rank correlation of 0.38 and 0.42 in asynchronous and S phase respectively (Figure 2B). These correlations further indicate that the association of Upf1 with gene loci primarily depends on their transcription, both in S phase cells and normal asynchronously growing cells.

The correlation between Upf1 and Pol II enrichment signals is visually apparent on the genome browser at some arbitrarily chosen genes that are associated with Upf1 based on the ChIP-chip data analysis: *pma1*, *met26*, *ght5*, *mug106* and *tpi1* (Supplementary Figure S3A-S3E). The signals are uniform throughout most of the gene body for both Upf1 and Pol II at these loci. This pattern appears to be genome-wide as it is also observed on the metagene plots calculated based on ChIP-chip datasets (Supplementary Figure S4). However, there seems to be a lower signal at the start and end of genes for both Upf1 and Ser5 Pol II.

On the other hand, two other randomly chosen genes which show no Upf1 signals, *ada2* and *SPBC609.0*, also displayed no or minimal Pol II signals (Supplementary Figure S3F–G). One exception in this set is *mug106*, which despite having an apparent Upf1 signal throughout the gene, does not show a Ser5 Pol II signal according to the ChIP-chip data (Supplementary Figure S3D). However, there is some total Pol II signal throughout its length based on the Rpb3 ChIP-seq described below (genome browser profiles of total Pol II at *mug106* and the other six genes discussed are shown in Supplementary Figure S5). It is possible that the Pol II transcribing this gene is either not or low Ser5 phosphorylated. Conversely, there are also some highly transcribed genes that show no or little association with Upf1, for example, *gpm1*, a gene just downstream of *mug106* (Supplementary Figure S3D). The reason why Upf1 is not associated with these genes is yet to be determined.

With regard to mRNA expression levels, the selected genes range from *pma1*, one of the most highly expressed in *S. pombe*, to *SPBC609.01* and *mug106* which are expressed at a much lower level in standard growth conditions (Supplementary Figure S6). The association of Upf1 with all these genes was further examined by ChIP-qPCR in an independent experiment using the Flag-tagged *upf1* strain (Figure 2C). The levels of Ser2 Pol II at these genes was also assessed by ChIP-qPCR (Figure 2D) and these correlate with Upf1 signals at most of the gene regions (Figure 2C right panel shows correlation of Upf1 with Ser2 values taken from Figure 2D; Figure 2D right panel displays the correlation of Ser2 level with itself, showing the signals’ ranking; replicates of these two experiments and error bars are shown in Supplementary Figure S7). In summary, although there are exceptions, the degree of Upf1 association with active genes correlates genome-wide with Pol II loading and RNA levels.

### Upf1 does not copurify with Pol II

**Figure 3.**
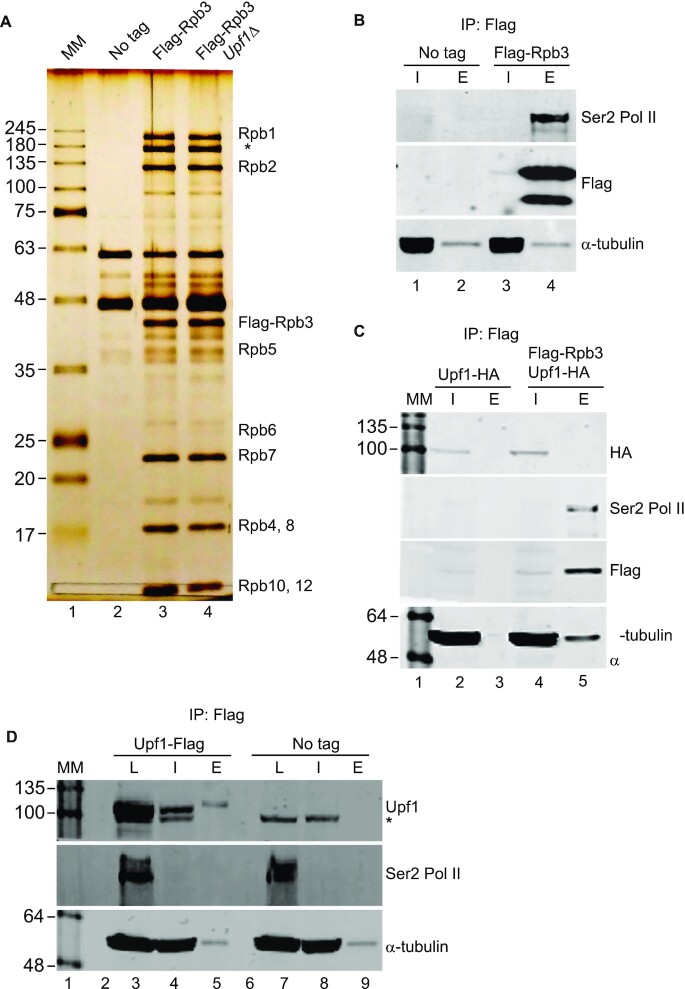
Upf1 does not copurify with Pol II. (**A**) Silver stained gel of affinity purified Pol II protein complex using Flag tagged Rpb3 (labelled IP: Flag). Three strains used for this assay were non-tagged wild-type (lane 2), Flag tagged Rpb3 (lane 3) and Flag tagged Rpb3 in a *upf1*Δ strain (lane 4). Molecular kDa marker (MM) was loaded in the lane 1. Different subunits of the Pol II complex copurifying with Flag tagged Rpb3 are labelled on the right hand side—note the two bands at the top of the gel correspond to Rpb1, the lower band (asterisk) was confirmed to be also a Rpb1 species by mass spectrometry (data not shown) and could be a cleavage product produced during the purification. (**B**) Western blots for Ser2 Pol II, Flag-Rpb3 and α-tubulin (as indicated on the right) of protein extract or purified fractions from non-tagged wild-type control (lanes 1 and 2) and Flag-Rpb3 (lanes- 3 and 4) strains. Where I is input and E eluted Flag purified proteins. (**C**) Western blots for the 4 proteins indicated on the right of each panel from protein extracts or purified fractions from Upf1-HA (lanes 2 and 3) and Upf1-HA + Flag-Rpb3 strains (lanes 4 and 5). Where I is input and E- eluted Flag purified proteins. (**D**) Western blots for the three proteins indicated of extract/purified fractions from Upf1-Flag (lanes 3, 4 and 5) and non-tagged WT (lanes 7, 8 and 9) strains. Where L is total lysate (prior DNase treatment and centrifugation clearing), I is input (centrifugation supernatant), E is eluted Flag purified proteins. The asterisks indicate a non-specific cross-reacting band.

The RNase sensitivity of the ChIP signal indicates that Upf1 is primarily associated with the nascent transcript. To investigate other potential interactions between the two, we examined whether Upf1 copurifies with Pol II. Pol II was purified from a strain encoding the Rpb3 subunit of Pol II functionally tagged with a single copy of Flag (41). In a similar strain Upf1 was also tagged with HA. The Pol II purification procedure (Materials and Methods) was validated by silver-stained SDS-PAGE of the Flag elution fraction, which confirmed co-purification of the expected bands corresponding to Rpb1 (two top bands) and most of other Pol II subunits (Figure 3A). There were no apparent experimentally reproducible changes in the protein banding pattern of the Pol II complex purified from wild-type and *upf1Δ* (Figure 3A). The identity of the putative Rpb1 bands was confirmed by mass spectrometry (not shown) and by western blotting (Figure 3B). However, there was no evidence of a putative Upf1 band in the Pol II fraction (Figure 3A). Furthermore, Upf1 could not be detected in the Pol II elution fraction by western blotting of purified Pol II from the Flag-Rpb3/Upf1-HA double tagged strain (Figure 3C, lane 5). There is also no evidence of Pol II copurifying with Upf1 in the reverse experiment using a strain carrying Flag-tagged Upf1 (Figure 3D). These data thus show no evidence of a direct stable interaction of Upf1 with Pol II and that, as the purification was performed under conditions that should keep the nascent transcript intact, the association of Upf1 with the nascent mRNA should be dynamic and it is lost during the purification.

### There are changes in Pol II loading and Ser2 phosphorylation at active genes in *upf1Δ*

**Figure 4.**
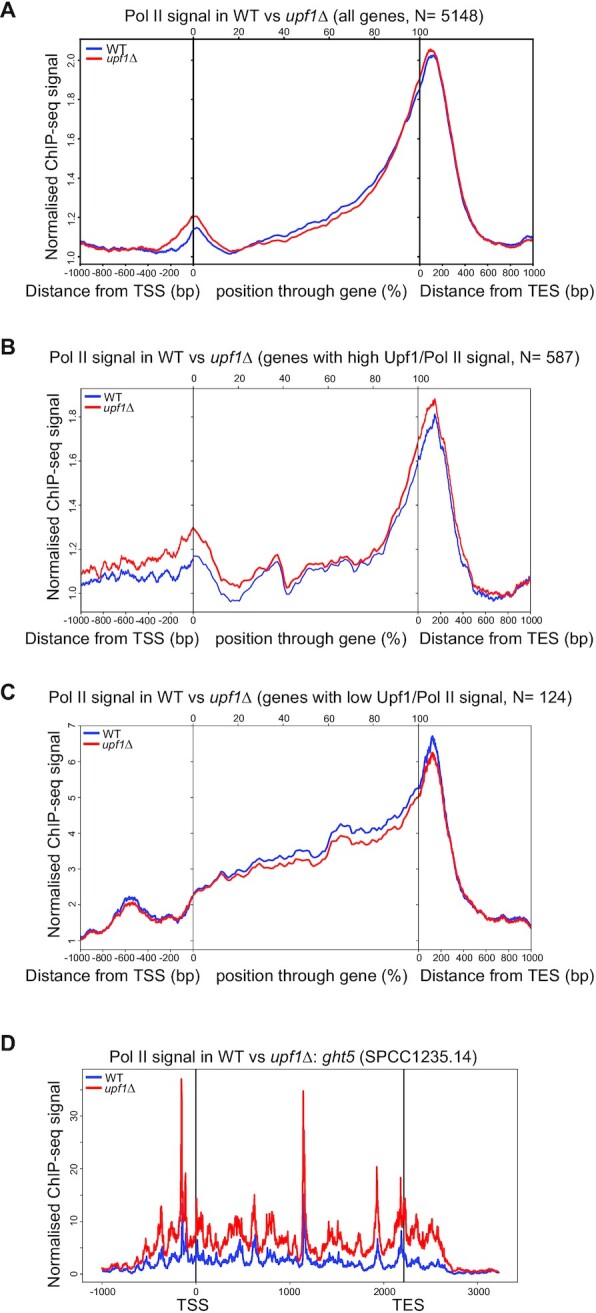
Increased Pol II loading at TSS and TES in *upf1*Δ. (**A**) Metagene analysis of genome-wide binding of total-Pol II from Rpb3 ChIP-seq datasets from wild-type (WT) and *upf1*Δ strains. The averaged profiles for all the genes are displayed. The analysed region covers the sequence 1000 bp upstream of the TSS, the ORF region and 1000 bp downstream of the TES. (**B**) Metagene analysis as in A of genes having high Upf1-to-Ser5 Pol II signal (group 1, as described in Results section). (**C**) Metagene analysis as in A of genes having low Upf1-to-Ser5 Pol II signal (group 2, as described in Results section). (**D**) Profiles of total-Pol II ChIP-seq signals at *ght5* in WT and *upf1*Δ (termed KO in this panel) strains. Units on x-axis are base pairs relative to the gene TSS and TES sites.

Next, we examined whether there were changes in the genome-wide distribution of total Pol II by ChIP-seq of Flag-tagged Rpb3 in *upf1Δ* and wild-type strains. The ChIP-seq data were processed and metagene plots were produced by taking the coverage signal values from 1kb upstream of the transcription start site (TSS), to 1kb downstream of the transcript end site (TES) and for the gene body of all annotated protein coding genes (Material and Methods). This analysis indicated the expected Pol II gene loading for *S. pombe* in both wild-type or *upf1Δ* with the characteristic increased signal of Pol II downstream of the TES (Figure 4A). This 3′ end skewed total Pol II metagene pattern, which differs from that seen in other organisms, has been previously discussed in *S. pombe* (40,42). This pattern is observed at many individual highly transcribed genes (several examples are shown in Supplementary Data File 1).

This initial analysis indicates that at most genes there are only minor changes in total Pol II loading between wild-type and *upf1Δ* (Figure 4A), other than a possible slightly increased signal proximal to the TSS. However, there are a few specific genes, among those strongly associated with Upf1 in wild-type cells, that show increased total Pol II signal in *upf1Δ*, either in the gene body, or around the TES or downstream of the TES (Supplementary Data File 1). To explore the significance of these Pol II changes in *upf1Δ*, we divided genes in three groups based on different levels of Upf1 relative to Pol II ChIP-chip signal: 1) high Upf1 signal relative to Pol II (587 genes), 2) low Upf1 signal relative to Pol II (124 genes) or 3) similar levels of Upf1 and Pol II (4177 genes—see Materials and Methods and the scatter plot in Supplementary Figure S8A for how the groups were defined). The genes in these different groups are listed in Supplementary Table S4.

Notably, the metagene analysis of these groups shows that transcription of these genes with high Upf1-to-Pol II signal are affected most by Upf1 deletion. In this group there is an apparently increased Pol II signal at both TSS and TES downstream proximal regions. Conversely, the genes with low Upf1-to-Pol II signals, which are essentially mid-to-high expressed genes to which Upf1 is not or poorly associated, show no evidence of Pol II build-up at neither TSS proximal regions and downstream of the TES (Figure 4B versus C). Instead in the largest group, corresponding to genes with medium Upf1-to-Pol II signal, only the TSS-proximal Pol II build-up is apparent when all genes were included irrespectively of Upf1 association as expected (Supplementary Figure S8B versus Figure 4A). A striking example of increased Pol II loading in *upf1Δ* is seen at *ght5* (Figure 4D), which is one of the genes in group 1 with high Upf1-to-Pol II signal and also one of the genes that was validated for Upf1 and Pol II association by ChIP-qPCR. The *ght5* gene is also mis-regulated in *upf1Δ* as discussed further below.

**Figure 5.**
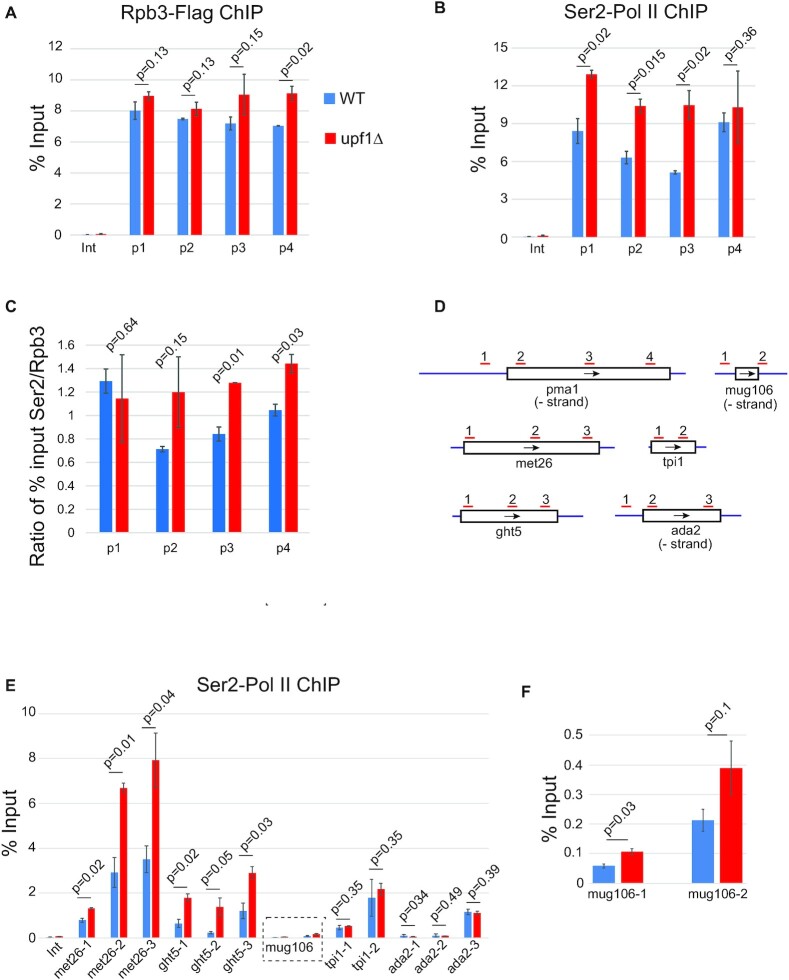
Increased Ser2 Pol II signal on active genes. (**A**) qPCR of Flag-Rpb3 ChIP signal on *pma1* in WT and *upf1*Δ strains. Results are shown as percentage of input enrichment for the average of two independent biological samples with three replicates each (mean ± SEM), the signal at an intergenic (Int) region is shown as background control for each replicate. Statistical analysis of differential Flag-Rpb3 accumulation was performed using one-tailed Student's t-test. *P*-values are shown above each primer pair. (**B**) Ser2 Pol II ChIP signal on *pma1* in WT and *upf1*Δ strains. Statistical analysis of differential Ser2 Pol II accumulation was calculated as mentioned above. (**C**) Ratios of Ser2 to Rpb3 signals shown in B and A respectively. Error bars and statistical test were calculated as above. (**D**) Schematic of tested genes with locations of qPCR primers indicated. (**E**) Flag-Rpb3 ChIP signal on 5 genes in WT and *upf1*Δ strains. (**F**) Flag-Rpb3 ChIP signal on *mug106* gene. Statistical analysis of differential Flag-Rpb3 accumulation was calculated as mentioned above.

Finally, higher Ser2 signal is also seen by ChIP-qPCR in *upf1Δ* at all mid-to-highly active genes that were examined. These are the same genes initially selected as showing high Upf1 ChIP-chip signal in wild-type that were verified by ChIP-qPCR. One of the genes is *pma1*, which although does not show significant changes in total Pol II loading in *upf1Δ*, with the possible exception of a small increase in the 3′ proximal region (Figure 5A). The levels of Ser2 Pol II signal are increased throughout the gene barring the 3′ proximal region (Figure 5B). Increased Ser2 signal is significant also when normalised by total Pol II at two of the four regions examined, including the 3′ proximal region (Figure 5C). Increased Ser2 signals are also seen at *met26* and *ght5* (Figure 5E). There could also be a small Ser2 signal increase at the low transcribed *mug106* gene (Figure 5F).

### There is a significant overlap between genes bound by Upf1 and genes differentially expressed in *upf1*Δ cells

**Figure 6.**
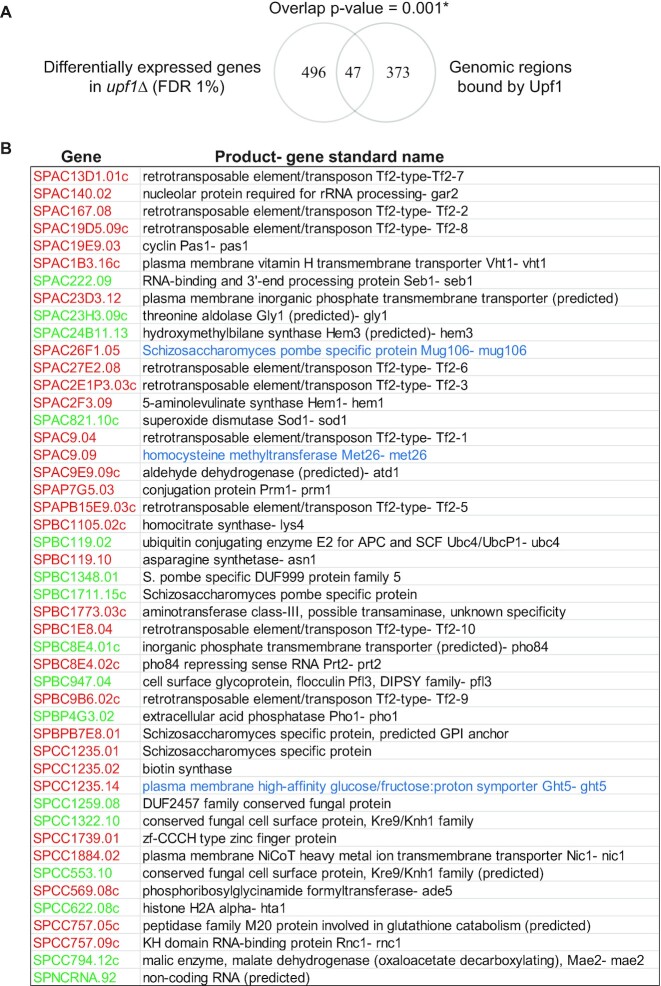
Significant overlap between genes bound by Upf1 and genes differentially expressed in a *upf1*Δ strain. (**A**) Venn diagram showing the overlap between the genes/genomic regions strongly associated with Upf1 (420, identified using the *P*-value cut off of 10^–4^ in the MAT software) and differentially expressed genes in *upf1*Δ (543). The asterisk indicates that the *P*-value of this overlap is either 0.001 based on random sampling, or of 0.006 based on a Fisher's exact hypergeometric test (Material and Methods). (**B**) List of the 47 overlapping genes. Genes highlighted in red are up-regulated and genes highlighted in green are down-regulated. Genes which were tested and show increased Ser2 CTD phosphorylation are shown with their full names in blue.

To explore further whether Upf1 may have some function in the expression of the genes to which it is associated, we compared these genes with genes that are differentially expressed in *upf1Δ*. We analysed a previous RNA microarray dataset (35), and used significance analysis of microarrays (SAM) with a 1% FDR to find differentially expressed genes between the wild-type and *upf1Δ* samples (Materials and Methods). We identified a total of 543 genes differentially expressed between the wild-type and *upf1Δ* using these parameters. Of these, 159 show reduced mRNA levels, whereas almost double this number (384) of genes show increased mRNA levels in *upf1Δ* (Figure 6A and Supplementary Table S5). Of the 543 differentially expressed genes, 47 are also strongly bound by Upf1 according to our ChIP-chip data (Figure 6B; red and green codes indicate 31 up and 16 down regulated genes, respectively). Based on the number of different genes represented on the microarrays we calculated the *P*-value of this overlap to be either 0.001 and 0.006 depending on the statistical test used. The individual *P*-values of the overlap between up-regulated genes and Upf1 associated genes was of 0.05, whereas the overlap between down-regulated and Upf1 associated genes was of 0.03 (Material and Methods). Notably, two of these genes are *met26* and *ght5*, which as described above show increased Ser2 signal, and in the case of *ght5*, also increased total Pol II in *upf1Δ* compared to wild-type. Both genes are upregulated in *upf1Δ* (names in blue characters in Figure 6B). Note that although *pma1* is not mis-regulated, an antisense ncRNA gene (SPNCRNA.92) located in the 5′ UTR of *pma1* is downregulated in *upf1Δ* (Figure 6B). This ncRNA gene is strongly associated with Upf1 according to the ChIP-chip data (Figure 1B). The RNA level of several Tf2 retrotransposons, which might also be associated with Upf1, as discussed, are also increased in *upf1Δ* according to both the microarray dataset and qRT-PCR validation (Figure 6B and Supplementary Figure S1A, S1B). Notably, one of the Tf2 elements (Tf2-5, SPAC2E1P3.03c) also shows increased total Pol II loading in *upf1Δ* cells compared to wild-type, including in the non-repetitive 3′ end region, suggesting that Tf2-5 may be transcriptionally up-regulated in these cells (Supplementary Data File 2). The increased level of the *ght5* transcript was confirmed by RT-qPCR (Supplementary Figure S9A), showing ght5 transcript stability is not enhanced in *upf1Δ* as compared to wild-type, based on its decay profile following transcription inhibition with 1,10-phenanthroline (Supplementary Figure S9B).

### Upf1 deficient cells are hypersensitive to 6-azauracil

**Figure 7.**
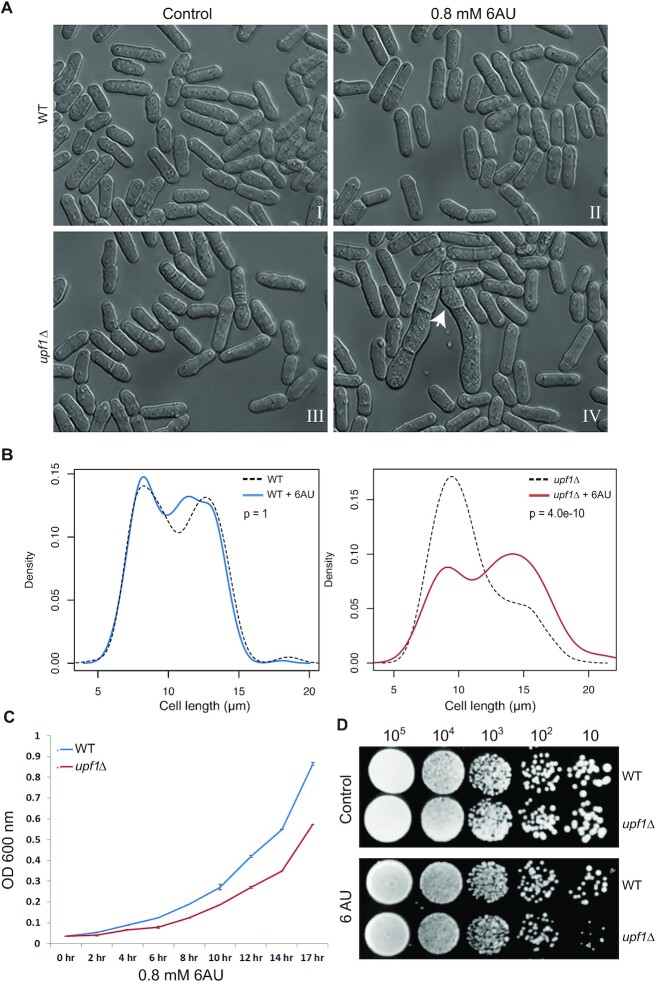
Upf1 deficient cells are hypersensitive to 6-Azauracil. (**A**) Photomicrographs of WT and *upf1*Δ *S. pombe* cells growing in standard YES (left panel) or YES + 0.8 mM 6AU (right panel) medium. Long, unseparated chains of cells are indicated with a white arrow. (**B**) Growth assay curves of WT and *upf1*Δ cells grown in YES + 0.8 mM 6AU media. X-axis shows the different time points at which OD_600_ was measured. (**C**) Serial dilution colony growth assay of the WT and *upf1*Δ cells, spotted on YES and YES + 0.8 mM 6AU plates. (**D**) Western blotting of total S. pombe protein extract from cells at different time points from a growing culture (the OD_600_ of the culture at each point is indicated above). The top half of the membrane was incubated with a Ser2 Pol II antibody and the bottom half with an alpha-tubulin antibody (as loading control). Lanes 1 and 10 show the same protein molecular weights marker (values are in kilodaltons).

To explore further whether Upf1 has a role in Pol II transcription, we examined whether *upf1*Δ cells are hypersensitive to 6-azauracil (6AU). It has previously been reported that strains carrying mutations in components of the RNA polymerase II transcription elongation machinery are hypersensitive to 6AU (43,44). 6AU is an inhibitor of enzymes that are involved in nucleotide biosynthesis; 6AU treatment leads to nucleotide depletion and hence can diminish transcription elongation (45). When grown in 0.8 mM 6AU, frequent morphology and septation defects were observed in *upf1*Δ but not in the wild-type strain, with the appearance of long unseparated chains of cells (Figure 7A, panels II versus IV). Cells longer than 15 μm are very rarely observed in the wild-type strain regardless of 6AU treatment (Figure 7B, left panel). However, these are significantly more frequent in the presence of 6AU in *upf1*Δ (Figure 7B, right panel); whilst there is no significant difference in median cell sizes between wild-type and *upf1*Δ untreated strains (the density plots shown in Figure 7B correspond to the size distributions of 300 cells in each of the four groups - the frequency of different cell size classes in these groups and statistical comparison between all pair combinations are reported in Supplementary Figure S10). These long cell phenotypes of 6AU in *upf1*Δ are similar to those previously described for the elongation mutants referred above. These appear 2 h after addition of the drug under standard growth conditions and persisted at all later time points examined up to 3 h (not shown). The *upf1Δ* strain also shows a slow growth phenotype in the presence of 6AU, in both liquid cultures and on agar plates (Figure 7C and D).

### Rbp1 shows slower gel mobility in *upf1Δ*

It was also examined whether there are changes in Pol II phosphorylation by western blotting of whole-cell lysates of cells taken from a growing culture at different intervals. A Ser2 specific antibody was used, which is expected to detect Ser2 phosphorylated CTD of Pol II largest subunit Rpb1. The slowest/top migrating species that this antibody detects should represent Rpb1 with a fully phosphorylated CTD. Notably, it appears that the largest band that the Ser2 antibody detects is sharper and slightly upward shifted in *upf1Δ* compared to wild-type (Supplementary Figure S11A, left panel), instead there is no apparent difference in mobility of the band detected by the antibody for the unphosphorylated CTD (8WG16) (Supplementary Figure S11A, right panel). This mobility shift was examined in cells from growing cultures at different OD_600_, and seems most apparent in cells at fastest growing stage of the culture (Supplementary Figure S11B, lanes 4 versus 5, OD_600_ 0.5, – unless otherwise specified, all the experiments described in this study, including that in Supplementary Figure S11A, were performed with culture at ∼OD_600_ 0.5). The overall cellular level of Ser2 seems lower in *upf1Δ* barring in the densest culture examined (OD_600_ 1, lanes 8–9). There might also be less of the cross-reacting faster migrating bands in *upf1Δ*; these could represent Rpb1 cleavage products of different sizes. Consistent with this interpretation, the largest of these products is also prominent in the SDS-PAGE of affinity purified Pol II fractions and its identity was validated by mass-spectrometry (Figure 3A, indicated by the asterisk). These data indicate that Pol II and CTD phosphorylation might be abnormal in *upf1Δ*, particularly in fast growing cells.

## DISCUSSION

The RNA helicase Upf1 has been mainly studied for its cytoplasmic role in NMD in *S. pombe* as well as in other model eukaryotes. In contrast to this broadly accepted view, we provide evidence that Upf1 is associated genome-wide with active genes in *S. pombe*. The association is mostly with protein-coding genes, RNase sensitive and positively correlates with Poll II loading as well as mRNA expression levels at most genes.

Apart from the start and to a lesser extent the end of genes, Upf1 association is uniform along gene bodies. The pattern is similar to that of the Ser5 ChIP-chip signal. This pattern could suggest that Upf1 recruitment to transcription sites might not be driven by its RNA binding, as a gradual increase in proportion to the length of the nascent transcript should be observed. This conclusion is consistent with our observation that there are highly transcribed genes that are not or low associated with Upf1 and reversely, there are low expressed genes to which Upf1 is strongly associated. A possible explanation is that Upf1 might associate with a protein component of the nascent mRNP rather than the pre-mRNA. In this scenario the association might be uniform along the gene because the nascent mRNP has only one copy of the component to which Upf1 binds—for example if Upf1 were to be recruited by regulated association with the cap binding complex.

Although only minor changes in total Pol II loading can be detected in *upf1*Δ cells when all protein coding genes are analysed together (Figure 4A), an increased Pol II signal at both TSS and TES downstream proximal regions was detected at a large group of genes characterised by having high Upf1-to-Pol II signal in wild-type (Figure 4B). Conversely, there is no evidence of Pol II build-up at neither TSS or TES proximal regions in the smaller group of well-expressed genes with low or no Upf1 ChIP signal (Figure 4C). Additionally, at a number of genes with which Upf1 clearly associates to in wild-type (for example at *ght5* and at other genes, Figure 4D and Supplementary Data File 2, respectively), Pol II loading is clearly increased throughout the transcribed region. These observations suggest that Upf1 affects transcription of the genes to which it is more strongly associated.

Notably, an increased Ser2 signal in *upf1*Δ was also detected at several active genes examined by ChIP-qPCR (*pma1*, *met26* and *ght5;* Figure 5). This was also confirmed for *pma1* by quantification of Ser2/total Pol II ratio. Whilst the higher Ser2 signal might be a consequence of increased Pol II loading at some genes (like at *ght5* for example), at others it might be primarily due to Ser2 CTD hyperphosphorylation. Consistent with hyperphosphorylation, the band corresponding to Ser2 phosphorylated Pol II (Rpb1) but not that corresponding to unphosphorylated Rpb1 migrates slower in *upf1*Δ compared to wild-type. It is therefore likely that more of the CTD repeats are phosphorylated in wild-type compared to *upf1*Δ. Ser2 hyperphosphorylation has previously been linked to slow transcription elongation in mammalian cells (46).

In view of these changes in Pol II loading and Ser2 phosphorylation, the possibility that Pol II elongation is altered in *upf1*Δ cannot be ruled out. Specifically, *upf1*Δ shows apparent 6AU hypersensitivity (Figure 7), which is a characteristic of transcription elongation *S. pombe* mutant strains (43). Additionally, an *S. pombe* strain carrying a mutant Pol II with reduced elongation rate is also hypersensitive to 6AU (47). Perhaps the role of Upf1 in transcription is more important in conditions of stress; exemplified by the nucleotide depleting conditions that the drug 6AU induces. Alternatively, it is plausible that CTD hyperphosphorylation is a consequence or a functional output of some yet unknown broad compensatory mechanism that maintains almost-normal transcription elongation rate at most genes in *upf1Δ*. Whether this putative compensatory mechanism relates to the mRNA decay dependent transcription adaptation phenomenon recently described in zebrafish, remains a possibility to be investigated in future studies (48).

**Figure 8.**
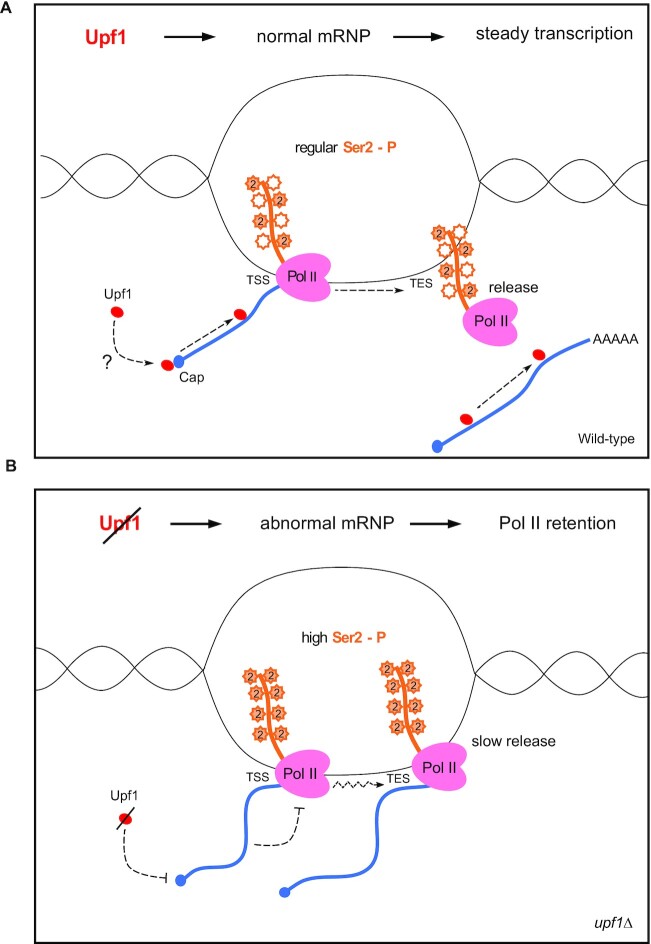
Upf1 controls transcription by association with the nascent mRNA. (**A**) Schematic of a site of transcription in wild-type cells. Upf1 (red) associates with nascent mRNA (blue line) by binding to a component of the mRNP for example, the cap-binding complex (blue dot). Upf1 scanning of the nascent mRNA results in a normal mRNP configuration, normal level of Ser2 phosphorylation (filled/non-filled orange stars), steady transcription elongation and termination. (**B**) Schematic of a site of transcription in *upf1Δ* cells. Absence of Upf1 results in abnormal mRNP configuration (twisted blue line), Ser2 hyperphosphorylation (filled orange starts), possible defective elongation (depicted by dashed zigzag arrow) and termination, and Pol II retention. This can occur either at premature termination sites downstream of the TSS or at normal transcription termination sites, downstream of the TES.

In summary, the data we have discussed indicate that Upf1 interacts with the nascent transcript, playing a role in transcription-coupled processes in *S. pombe*. We envisage that Upf1 may take part in some feedback system between the formation of the nascent mRNP, Pol II CTD phosphorylation and transcription (Figure 8). The increased Pol II signal downstream of the TSS could be explained by either defects in Pol II elongation leading to increased pausing or by defects in premature termination and Pol II release from these regions (49). The increase downstream of the TES could be due to similar mechanisms operating during normal termination or Pol II release. This is consistent with previous bioinformatic analyses that concluded human UPF1 interacts with DOM3Z, the homologue of yeast Rai1 (Rat-interacting protein 1), a protein that modulates the 5′→ 3′ exonuclease activity of Rat1/XRN2 during transcription termination (22).

The data we have presented are in agreement with the observations in *Drosophila* (27). Taken together, the findings from these two highly divergent organisms predict that the roles of Upf1 in transcription-coupled processes are conserved in eukaryotes. Species-specific differences are likely though. For example, apart from the slightly increased Pol II loading at TSS-proximal sites, which was also observed in *Drosophila*, there was no obvious evidence of increased Pol II loading downstream of TES and Ser2 phosphorylation in Upf1 depleted cells in this organism (50).

Furthermore, these data re-prompt questioning the fields’ communally accepted syllogism that transcripts whose levels are increased in Upf1 depleted cells are therefore likely to be NMD targets. This might not always be a valid explanation: it has previously been argued that only a fraction of these increases might actually be a direct consequence of cytoplasmic mRNA stabilization (8,10,51,52). Here, we have shown that there is overlap between genes associated with Upf1 and genes that have increased mRNA levels in *upf1*Δ. Such increases in mRNA levels would have, directly or indirectly, previously been attributed to NMD suppression in the cytoplasm. This explanation could be ruled out for *ght5* mRNA as it is not more stable in *upf1*Δ than in wild-type. However, it cannot be ruled out for other genes. It is plausible that the increased cellular mRNA levels might be caused by the absence of Upf1 from the transcription site of an affected gene, resulting in its abnormal expression. Some of the affected genes like *ght5* are apparently transcriptionally up-regulated in *upf1Δ*. Whether the apparent up-regulation of these set of genes is a direct consequence of the absence of Upf1 from their transcription sites, or indirectly, due to mis-expression of transcription regulators involved in their expression or due to some transcription adaptation mechanism remains to be addressed.

## DATA AVAILABILITY

The ChIP-chip and ChIP-seq datasets as well as all associated metadata files are available from a Gene Expression Omnibus (GEO) SuperSeries record: GSE169425 -https://www.ncbi.nlm.nih.gov/geo/info/linking.html.

The description of the bioinformatics pipelines used, custom-made scripts for correlation and metagene plots, raw data files and processed data tables are available at the GitHub repository: https://github.com/Brogna-Lab/PombeUpf1.

## Supplementary Material

gkab1249_Supplemental_Files

## References

[1] Goetz A.E. , Wilkinson M. Stress and the nonsense-mediated RNA decay pathway. Cell. Mol. Life Sci. 2017; 74:3509–3531. 28503708 10.1007/s00018-017-2537-6 PMC5683946

[2] Karousis E.D. , Nasif S., Muhlemann O. Nonsense-mediated mRNA decay: novel mechanistic insights and biological impact. Wiley Interdiscip. Rev. RNA. 2016; 7:661–682. 27173476 10.1002/wrna.1357 PMC6680220

[3] Hug N. , Longman D., Caceres J.F. Mechanism and regulation of the nonsense-mediated decay pathway. Nucleic Acids Res. 2016; 44:1483–1495. 26773057 10.1093/nar/gkw010 PMC4770240

[4] He F. , Jacobson A. Nonsense-mediated mRNA decay: degradation of defective transcripts is only part of the story. Annu. Rev. Genet. 2015; 49:339–366. 26436458 10.1146/annurev-genet-112414-054639 PMC4837945

[5] Fatscher T. , Boehm V., Gehring N.H. Mechanism, factors, and physiological role of nonsense-mediated mRNA decay. Cell. Mol. Life Sci. 2015; 72:4523–4544. 26283621 10.1007/s00018-015-2017-9 PMC11113733

[6] Lykke-Andersen S. , Jensen T.H. Nonsense-mediated mRNA decay: an intricate machinery that shapes transcriptomes. Nat. Rev. Mol. Cell Biol. 2015; 16:665–677. 26397022 10.1038/nrm4063

[7] Kim Y.K. , Maquat L.E. UPFront and center in RNA decay: UPF1 in nonsense-mediated mRNA decay and beyond. RNA. 2019; 25:407–422. 30655309 10.1261/rna.070136.118 PMC6426291

[8] Brogna S. , Wen J. Nonsense-mediated mRNA decay (NMD) mechanisms. Nat. Struct. Mol. Biol. 2009; 16:107–113. 19190664 10.1038/nsmb.1550

[9] Neu-Yilik G. , Raimondeau E., Eliseev B., Yeramala L., Amthor B., Deniaud A., Huard K., Kerschgens K., Hentze M.W., Schaffitzel C. et al. Dual function of UPF3B in early and late translation termination. EMBO J. 2017; 36:2968–2986. 28899899 10.15252/embj.201797079 PMC5641913

[10] Brogna S. , McLeod T., Petric M. The meaning of NMD: translate or perish. Trends Genet. 2016; 32:395–407. 27185236 10.1016/j.tig.2016.04.007

[11] Saikrishnan K. , Powell B., Cook N.J., Webb M.R., Wigley D.B. Mechanistic basis of 5′-3′ translocation in SF1B helicases. Cell. 2009; 137:849–859. 19490894 10.1016/j.cell.2009.03.036

[12] Singleton M.R. , Dillingham M.S., Wigley D.B. Structure and mechanism of helicases and nucleic acid translocases. Annu. Rev. Biochem. 2007; 76:23–50. 17506634 10.1146/annurev.biochem.76.052305.115300

[13] Fiorini F. , Bagchi D., Le Hir H., Croquette V. Human Upf1 is a highly processive RNA helicase and translocase with RNP remodelling activities. Nat. Commun. 2015; 6:7581. 26138914 10.1038/ncomms8581 PMC4506499

[14] Chakrabarti S. , Jayachandran U., Bonneau F., Fiorini F., Basquin C., Domcke S., Le Hir H., Conti E. Molecular mechanisms for the RNA-dependent ATPase activity of Upf1 and its regulation by Upf2. Mol. Cell. 2011; 41:693–703. 21419344 10.1016/j.molcel.2011.02.010

[15] Czaplinski K. , Weng Y., Hagan K.W., Peltz S.W. Purification and characterization of the Upf1 protein - a factor involved in translation and messenger RNA degradation. RNA. 1995; 1:610–623. 7489520 PMC1369305

[16] Bhattacharya A. , Czaplinski K., Trifillis P., He F., Jacobson A., Peltz S.W. Characterization of the biochemical properties of the human Upf1 gene product that is involved in nonsense-mediated mRNA decay. RNA. 2000; 6:1226–1235. 10999600 10.1017/s1355838200000546 PMC1369996

[17] Franks T.M. , Singh G., Lykke-Andersen J. Upf1 ATPase-dependent mRNP disassembly is required for completion of nonsense- mediated mRNA decay. Cell. 2010; 143:938–950. 21145460 10.1016/j.cell.2010.11.043 PMC3357093

[18] Mendell J.T. , ap Rhys C.M., Dietz H.C. Separable roles for rent1/hUpf1 in altered splicing and decay of nonsense transcripts. Science. 2002; 298:419–422. 12228722 10.1126/science.1074428

[19] Ajamian L. , Abel K., Rao S., Vyboh K., Garcia-de-Gracia F., Soto-Rifo R., Kulozik A.E., Gehring N.H., Mouland A.J. HIV-1 recruits UPF1 but excludes UPF2 to promote nucleocytoplasmic export of the genomic RNA. Biomolecules. 2015; 5:2808–2839. 26492277 10.3390/biom5042808 PMC4693258

[20] Azzalin C.M. , Lingner J. The human RNA surveillance factor UPF1 is required for S phase progression and genome stability. Curr. Biol. 2006; 16:433–439. 16488880 10.1016/j.cub.2006.01.018

[21] Rehwinkel J. , Letunic I., Raes J., Bork P., Izaurralde E. Nonsense-mediated mRNA decay factors act in concert to regulate common mRNA targets. RNA. 2005; 11:1530–1544. 16199763 10.1261/rna.2160905 PMC1370837

[22] Varsally W. , Brogna S. UPF1 involvement in nuclear functions. Biochem. Soc. Trans. 2012; 40:778–783. 22817733 10.1042/BST20120052

[23] De S. , Varsally W., Falciani F., Brogna S. Ribosomal proteins' association with transcription sites peaks at tRNA genes in *Schizosaccharomyces pombe*. RNA. 2011; 17:1713–1726. 21757508 10.1261/rna.2808411 PMC3162336

[24] Bahler J. , Wu J.Q., Longtine M.S., Shah N.G., McKenzie A. 3rd, Steever A.B., Wach A., Philippsen P., Pringle J.R. Heterologous modules for efficient and versatile PCR-based gene targeting in *Schizosaccharomyces pombe*. Yeast. 1998; 14:943–951. 9717240 10.1002/(SICI)1097-0061(199807)14:10<943::AID-YEA292>3.0.CO;2-Y

[25] Matsuo Y. , Asakawa K., Toda T., Katayama S. A rapid method for protein extraction from fission yeast. Biosci. Biotechnol. Biochem. 2006; 70:1992–1994. 16926515 10.1271/bbb.60087

[26] Abruzzi K.C. , Lacadie S., Rosbash M. Biochemical analysis of TREX complex recruitment to intronless and intron-containing yeast genes. EMBO J. 2004; 23:2620–2631. 15192704 10.1038/sj.emboj.7600261 PMC449771

[27] Singh A.K. , Choudhury S.R., De S., Zhang J., Kissane S., Dwivedi V., Ramanathan P., Orsini L., Hebenstreit D., Brogna S. The RNA helicase UPF1 associates with mRNAs co-transcriptionally and is required for the release of mRNAs from transcription sites. Elife. 2019; 8:e41444. 30907728 10.7554/eLife.41444 PMC6447362

[28] Johnson W.E. , Li W., Meyer C.A., Gottardo R., Carroll J.S., Brown M., Liu X.S. Model-based analysis of tiling-arrays for ChIP-chip. Proc. Natl. Acad. Sci. U.S.A. 2006; 103:12457–12462. 16895995 10.1073/pnas.0601180103 PMC1567901

[29] Nicol J.W. , Helt G.A., Blanchard S.G. Jr, Raja A., Loraine A.E The Integrated Genome Browser: free software for distribution and exploration of genome-scale datasets. Bioinformatics. 2009; 25:2730–2731. 19654113 10.1093/bioinformatics/btp472 PMC2759552

[30] Dennis G. , ShermanB.T., HosackD.A., YangJ., GaoW., LaneH.C., LempickiR.A DAVID: Database for Annotation, Visualization, and Integrated Discovery. Genome Biol.2003; 4:P3.12734009

[31] Wilhelm B.T. , Marguerat S., Watt S., Schubert F., Wood V., Goodhead I., Penkett C.J., Rogers J., Bahler J. Dynamic repertoire of a eukaryotic transcriptome surveyed at single-nucleotide resolution. Nature. 2008; 453:1239–1243. 18488015 10.1038/nature07002

[32] Bolger A.M. , Lohse M., Usadel B. Trimmomatic: a flexible trimmer for Illumina sequence data. Bioinformatics. 2014; 30:2114–2120. 24695404 10.1093/bioinformatics/btu170 PMC4103590

[33] Langmead B. , Salzberg S.L. Fast gapped-read alignment with Bowtie 2. Nat. Methods. 2012; 9:357–359. 22388286 10.1038/nmeth.1923 PMC3322381

[34] Gallagher P.S. , Larkin M., Thillainadesan G., Dhakshnamoorthy J., Balachandran V., Xiao H., Wellman C., Chatterjee R., Wheeler D., Grewal S.I.S. Iron homeostasis regulates facultative heterochromatin assembly in adaptive genome control. Nat. Struct. Mol. Biol. 2018; 25:372–383. 29686279 10.1038/s41594-018-0056-2 PMC5936480

[35] Rodriguez-Gabriel M.A. , Watt S., Bahler J., Russell P. Upf1, an RNA helicase required for nonsense-mediated mRNA decay, modulates the transcriptional response to oxidative stress in fission yeast. Mol. Cell. Biol. 2006; 26:6347–6356. 16914721 10.1128/MCB.00286-06 PMC1592850

[36] Tusher V.G. , Tibshirani R., Chu G. Significance analysis of microarrays applied to the ionizing radiation response. Proc. Natl. Acad. Sci. U.S.A. 2001; 98:5116–5121. 11309499 10.1073/pnas.091062498 PMC33173

[37] Collart M.A. , Oliviero S. Preparation of yeast RNA. Curr. Protoc. Mol. Biol. 2001; Chapter 13:Unit13 12. 10.1002/0471142727.mb1312s23 18265096

[38] Fukuda M. , Asano S., Nakamura T., Adachi M., Yoshida M., Yanagida M., Nishida E. CRM1 is responsible for intracellular transport mediated by the nuclear export signal. Nature. 1997; 390:308–311. 9384386 10.1038/36894

[39] Hutten S. , Kehlenbach R.H. CRM1-mediated nuclear export: to the pore and beyond. Trends Cell Biol. 2007; 17:193–201. 17317185 10.1016/j.tcb.2007.02.003

[40] Daulny A. , Mejia-Ramirez E., Reina O., Rosado-Lugo J., Aguilar-Arnal L., Auer H., Zaratiegui M., Azorin F. The fission yeast CENP-B protein Abp1 prevents pervasive transcription of repetitive DNA elements. Biochim. Biophys. Acta. 2016; 1859:1314–1321. 27345571 10.1016/j.bbagrm.2016.06.009 PMC5391875

[41] Kimura M. , Suzuki H., Ishihama A. Formation of a carboxy-terminal domain phosphatase (Fcp1)/TFIIF/RNA polymerase II (pol II) complex in Schizosaccharomyces pombe involves direct interaction between Fcp1 and the Rpb4 subunit of pol II. Mol. Cell. Biol. 2002; 22:1577–1588. 11839823 10.1128/mcb.22.5.1577-1588.2002 PMC134712

[42] Knoll E.R. , Zhu Z.I., Sarkar D., Landsman D., Morse R.H. Role of the pre-initiation complex in Mediator recruitment and dynamics. Elife. 2018; 7:e39633. 30540252 10.7554/eLife.39633 PMC6322861

[43] Zhou H. , Liu Q., Shi T., Yu Y., Lu H. Genome-wide screen of fission yeast mutants for sensitivity to 6-azauracil, an inhibitor of transcriptional elongation. Yeast. 2015; 32:643–655. 26173815 10.1002/yea.3085

[44] Shaw R.J. , Reines D Saccharomyces cerevisiae transcription elongation mutants are defective in PUR5 induction in response to nucleotide depletion. Mol. Cell. Biol. 2000; 20:7427–7437. 11003640 10.1128/mcb.20.20.7427-7437.2000 PMC86296

[45] Exinger F. , Lacroute F. 6-Azauracil inhibition of GTP biosynthesis in *Saccharomyces cerevisiae*. Curr. Genet. 1992; 22:9–11. 1611672 10.1007/BF00351735

[46] Fong N. , Saldi T., Sheridan R.M., Cortazar M.A., Bentley D.L. RNA Pol II dynamics modulate co-transcriptional chromatin modification, CTD phosphorylation, and transcriptional direction. Mol. Cell. 2017; 66:546–557. 28506463 10.1016/j.molcel.2017.04.016 PMC5488731

[47] Yague-Sanz C. , Vanrobaeys Y., Fernandez R., Duval M., Larochelle M., Beaudoin J., Berro J., Labbe S., Jacques P.E., Bachand F. Nutrient-dependent control of RNA polymerase II elongation rate regulates specific gene expression programs by alternative polyadenylation. Genes Dev. 2020; 34:883–897. 32499400 10.1101/gad.337212.120 PMC7328516

[48] El-Brolosy M.A. , Kontarakis Z., Rossi A., Kuenne C., Gunther S., Fukuda N., Kikhi K., Boezio G.L.M., Takacs C.M., Lai S.L. et al. Genetic compensation triggered by mutant mRNA degradation. Nature. 2019; 568:193–197. 30944477 10.1038/s41586-019-1064-z PMC6707827

[49] Kamieniarz-Gdula K. , Proudfoot N.J. Transcriptional control by premature termination: a forgotten mechanism. Trends Genet. 2019; 35:553–564. 31213387 10.1016/j.tig.2019.05.005 PMC7471841

[50] Singh A.K. , Zhang J., Hebenstreit D., Brogna S. Evidence of slightly increased Pol II pausing in UPF1-depleted Drosophila melanogaster cells. MicroPubl. Biol. 2020; 2020:10.17912/micropub.biology.000319. PMC7704256 33274326

[51] Wen J. , Brogna S. Splicing-dependent NMD does not require the EJC in *Schizosaccharomyces pombe*. EMBO J. 2010; 29:1537–1551. 20360683 10.1038/emboj.2010.48 PMC2876954

[52] Wen J. , He M., Petric M., Marzi L., Wang J., Piechocki K., McLeod T., Singh A.K., Dwivedi V., Brogna S. An intron proximal to a PTC enhances NMD in Saccharomyces cerevisiae. 2020; bioRxiv doi:18 March 2020, preprint: not peer reviewed 10.1101/149245.

