# Supplementary material for: Genome-wide chromosomal association of Upf1 is linked to Pol II transcription in Schizosaccharomyces pombe
Source: Nucleic Acids Res. 2021 Dec 20;50(1):350–67. doi: 10.1093/nar/gkab1249 (PMC8754637; doi:10.1093/nar/gkab1249)
Supplement: gkab1249_Supplemental_Files [file gkab1249_supplemental_files.zip › De_et_al_Supplementary_data_sb2L1M2_hd3.pdf]

Supplementary Data

Genome-wide chromosomal association of Upf1 is linked to Pol II transcription in *Schizosaccharomyces pombe*

Sandip De, David M. Edwards, Vibha Dwivedi, Jianming Wang, Wazeer Varsally, Hannah L. Dixon, Anand K. Singh, Precious O. Owuamalam, Matthew T. Wright, Reece P. Summers, Md Nazmul Hossain, Emily M. Price, Marcin W. Wojewodzic, Francesco Falciani, Nikolas J. Hodges, Marco Saponaro, Kayoko Tanaka, Claus M. Azzalin, Peter Baumann, Daniel Hebenstreit and Saverio Brogna

Supplementary Table S1

S. pombe strains used in this study

WT	*h- ade6-M216 leu1-32 ura4-D18*	Current Genetics 1996 30(4):284-93.
WT	*h+ ade6-210 arg3D his3D leu1-32 ura4DS/E*	Current Genetics 1996 30(4):284-93.
Upf1-HA Cdc25-22	*h? cdc25-22 ade6-704 leu1-32 ura4-D18 upf1-3HA::kanMX6*	This study
Upf1:Flag	*h+ ade6-210 arg3D his3D leu1-32 ura4DS/E upf1:5flag::hphMX6*	This study
Flag-Rpb3	*h- flag-rpb3 ade6-M216 ura4-D18 leu1*	From Japanese National BioResource Project, strain JY741.
upf1Δ Flag-Rpb3	*h- flag-rpb3 ade6-M216 ura4-D18 leu1 upf1::hphMX6*	Mol Cell Biol. 2006 26(17): 6347–6356.
Flag-Rpb3 Upf1-HA	*flag-rpb3 upf1-3HA::kanMX6 ade6-M216 ura-D18 leu h-*	This study
Flag-Rpb3 *upf1Δ*	*h-flag-rpb3 ade6-M216 ura4-D18 leu 1 upf1::hphMX6*	This study

Supplementary Table S2

Primers used in this study

	Primer name	Sequence	Comments
Pma1	Pma1-p1-F	GTCTTCGTGATTGGGTCGAT	for radioactive PCRs
	Pma1-p1-R	GGGGTCACCATAGTGCTTGT	for radioactive PCRs
	Pma1-p2-F	ATCCCGTTTCCAAGAAGGTT	for radioactive PCRs
	Pma1-p2-R	GAGGATCGGAACAAGGCATA	for radioactive PCRs
	Pma1-p3-F	GTCTTTCCACCGTCATTGGT	for radioactive PCRs
	Pma1-p3-R	ACGGAGAACGGCAACAATAG	for radioactive PCRs
	Pma1-p4-F	GAAACTATAGGTTAATGGAAG	for radioactive PCRs
	Pma1-p4-R	GTTTCCTGCCGGCTTGTC	for radioactive PCRs
Act1	Act1-t1-F	GCTCAATGTTATCCGTTTCCG	for radioactive PCRs
	Act1-t1-R	GTAGTTGGTAAACGGTAAGTTATAACAC	for radioactive PCRs
	Act1-t2-F	GGAAGAAGAAATCGCAGCGT	for radioactive PCRs
	Act1-t2-R	CATATCATCCCAGTTGTTGACAATAC	for radioactive PCRs
	Act1-t3-F	GAAATGTGATGTTGATATTCGTAAAG	for radioactive PCRs
	Act1-t3-R	GCTCTCATCATACTCTTGCTTGG	for radioactive PCRs

Intergenic	Intergenic-RT-F	AGAGGCACATAGTAGGGGAACT	for radioactive PCRs
	Intergenic-RT-R	TCCCATCTCCCACTGTTAATTGA	for radioactive PCRs
Tf2	tf2	ACACCAACACAAACCCAAGCGA	For qPCRs. From 2035-2056 bp 0f ORF of tf2-1
	tf2	ACGGCTCCTACAGCGACATCT	For qPCRs. From 2165-2145 bp 0f ORF of tf2-1
tRNAMet	tRNAMet#1F	AAAAGAAAACGGTCAGGGAGG	For qPCRs. Pebernard, 2008; SPBTRNAMET.05
	tRNAMet#1R	GAGCCTCACCAGGAGCATTATAG	For qPCRs. Pebernard, 2008; SPBTRNAMET.05
tRNAlAla	tRNAAla#1R	CCTGCAAACGTATGTTACGTAAGG	For qPCRs. Pebernard, 2008
	tRNAAla#1F	TCCAATTATTAAGTGAATGCTCTCG	For qPCRs. Pebernard, 2008
tRNAAsn	tRNAAsnF	GGTCGGGTAGCATAGTTGGTT	For qPCRs. SPBTRNAASN.01
	tRNAAsnR	AGAAAACGGTCAGGGAGGGA	For qPCRs. SPBTRNAASN.01
Telomere	tel	TCA AAG TTG GCG ACG TTG CTG ATG	For qPCRs. Detect telomeric region; Rozenzhak S, 2010
	tel	AAG CAA TGT GTG GAG CAA CAG TGG	For qPCRs. Detect telomeric region; Rozenzhak S, 2011

Intergenic	Intergenic_R	GCGAAACCAGTATGGACGAT	for qPCRs
	Intergenic_F	AACGGGCAAATGTAAAGACG	for qPCRs
SPBC609.01	SPBC609.01-1F	AAGGGATGCAGACAACTCCA	for qPCRs
	SPBC609.01-1R	GGTTTCAAAGGCGTCAGGAA	for qPCRs
	SPBC609.01-2F	CACACTGAGCAACTTCTACCG	for qPCRs
	SPBC609.01-2R	TCAACAGCCAAAACAAAAGCC	for qPCRs
met26	met26-1F	CGTGAGTTGAAGAAGGCCAC	for qPCRs
	met26-1R	CAACACCTTGGGCCTTTTGA	for qPCRs
	met26-2F	AAGTTGCTTCTTCCTCCAGC	for qPCRs
	met26-2R	GCCGTCATTAGCAGCCTTAG	for qPCRs
	met26-3F	GGATGCTGACGTTGTTTCCA	for qPCRs
	met26-3R	AACAGGAGGAACACGAGGAG	for qPCRs
ght5	ght5-1F	TCATGTTGGTCTTCGTGTCC	for qPCRs
	ght5-1R	CGCGCCGAAGAATACGAATA	for qPCRs
	ght5-2F	CGCTACTTGGCCCGAAATTT	for qPCRs
	ght5-2R	CCCTCAAACACCTCGAAACC	for qPCRs
	ght5-3F	ATCTCTGGTGCTAAGCCCTG	for qPCRs
	ght5-3R	ACGTTTTCAAGATGAGCGGC	for qPCRs
mug106	mug106-1F	TCCTTTTCCACCTGCAAACG	for qPCRs
	mug106-1R	TTCATGTGCTGGATTGGGTT	for qPCRs
	mug106-2F	TCTCCCATTTTGCACGAGGA	for qPCRs
	mug106-2R	TCGCCTACTAACACCGGTAC	for qPCRs
tpi1	tpi1-1F	CGTTGGTGATGTCGAAACTGT	for qPCRs
	tpi1-1R	GTAGGCACCGTTCTTCTTGTC	for qPCRs
	tpi1-2F	CTGTCTGGGCCATTGGTACT	for qPCRs

	tpi1-2R	GTAGATGACACGGAGACCCT	for qPCRs
ada2	ada2-1F	TGTTCCAAAAGGCAACATGG	for qPCRs
	ada2-1R	TCTTACTGCCGAAGCGAATG	for qPCRs
	ada2-2F	CACCATCCGTCTCATCCGTA	for qPCRs
	ada2-2R	CTGCAATGTCAGCCCAGTTT	for qPCRs
	ada2-3F	CAGCGACTCCCACAATGTTT	for qPCRs
	ada2-3R	TGGAAGTCAGTGAAGCCGAT	for qPCRs
Pma1	qP4 R PMA1	GTCTTTCCACCGTCATTGGT	for qPCRs
	qP4 F PMA1	ACGGAGAACGGCAACAATAG	for qPCRs
	qP3 R PMA1	ATCCCGTTTCCAAGAAGGTT	for qPCRs
	qP3 F PMA1	GAGGATCGGAACAAGGCATA	for qPCRs
	qP2 R PMA1	GTCTTCGTGATTGGGTCGAT	for qPCRs
	qP2 F PMA1	GGGGTCACCATAGTGCTTGT	for qPCRs
	qP1 R PMA1	CTCTAGAACATACGTTATTTAATCTCGA	for qPCRs
	qP1 F PMA1	GTATTACCGACAATAGAAAAGGGG	for qPCRs
Upf1-HA Tagging	Upf1-F	TGTTACAATTATTTACACTTTGCAAATTGACGGCTTAATAACATATCAAGTTGTCTTTCCCGGATCCCCGGGTTAATTAA	
	Upf1-R	ATATCAACAAATAAAAGATATGTTGGCATTCGTAATTACAAGTAAGCAAATACTTATTAGAATTCGAGCTCGTTTAAAC	
Upf1-FLAG Tagging	Upf1_ctl_F	AACGTTGCATTGACTCGAGC	
	Upf1_ctl_R	GGGGATCCGTCGACCTGCAGCGTACGAGAACCTAGTAGGTTCGTCGA	
	Upf1_ctr_F	GTTTAAACGAGCTCGAATTCATCGATTTAATAAGTATTTGCTTACTTGTAATTACG	
	Upf1_ctr_R	ACTGGCTATCTGTTGTAATTGGC	
RpL32	RpL32 F	TGGATTGAAGGCTTTCCTAGTC	qRT-PCR
	RpL32 R	CAGAGACGTTACCAGCAATCT	qRT-PCR
18S rRNA	18S F	CAATTGGAGGGCAAGTCTGG	qRT-PCR
	18S R	GTCGACCAGGCTCAAAGTTC	qRT-PCR

Supplementary Table S3

List of Upf1-associated genes, provided as separate Excel file labelled Supplementary Table S3

Supplementary Table S4

List of genes having different Upf1 vs. Pol II ChIP-chip signal ratios, provided as separate Excel file labelled Supplementary Table S4

Supplementary Table S5

List of genes differentially expressed in *upf1*Δ, provided as separate Excel file labelled Supplementary Table S5

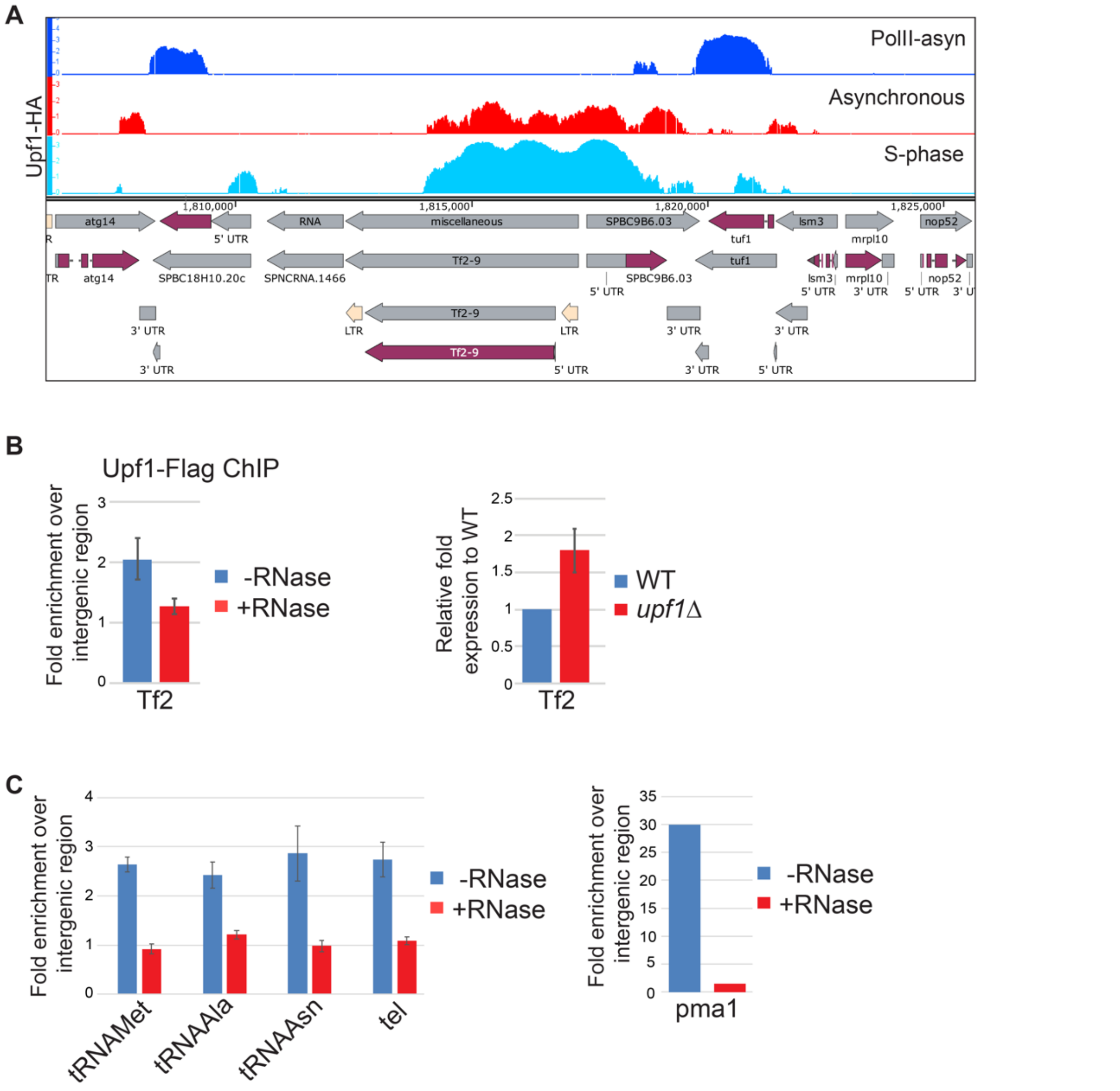

Supplementary Figure S1. Upf1 association to tRNA genes and transposable elements loci is RNase sensitive. A) IGB screenshot of ChIP-chip enrichments of Ser5 Pol II in asynchronous culture (top row), Upf1-HA in asynchronous culture (middle row) and in S-phase culture (bottom row) over Tf2-9 gene and its flanking region of *S. pombe*. Genes and genomic features are shown below. **B**) Left panel- Upf1-Flag qPCR-ChIP signal of a transposable element Tf2 sequence with and without RNase treatment. Right panel- expression of Tf2s in WT vs *upf1*Δ cells. **C**) Left panel- Upf1-HA ChIP signal on tDNAs and telomere. Right panel- Upf1-HA ChIP signal on *pma1* without and with the RNase treatment.

Figure S2

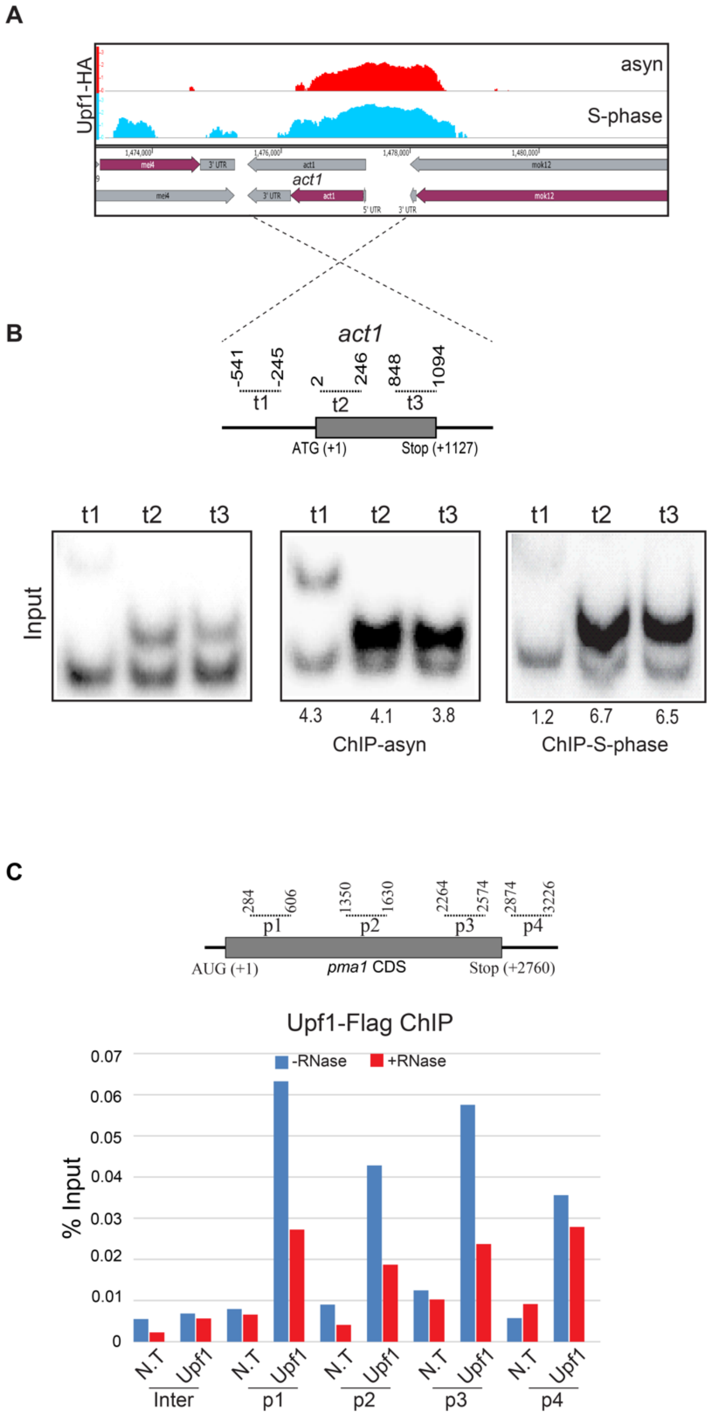

A
Upf1-HA
asyn
S-phase
act1
B
act1
-541
-245
2
246
848
1094
t1
t2
t3
ATG (+1)
Stop (+1127)
t1 t2 t3
Input
4.3 4.1 3.8
ChIP-asyn
1.2 6.7 6.5
ChIP-S-phase
C
284
606
1350
1630
2264
2574
2874
3226
p1
p2
p3
p4
AUG (+1)
pma1 CDS
Stop (+2760)
Upf1-Flag ChIP
-RNase
+RNase
% Input
0.07
0.06
0.05
0.04
0.03
0.02
0.01
0
N.T
Upf1
Inter
p1
p2
p3
p4

Supplementary Figure S2. Upf1 association to specific genes is RNase sensitive. A) IGB screenshot of ChIP-chip enrichments of Upf1-HA in asynchronous culture (top row) and in S-phase culture (bottom row) over *act1* gene and its flanking region. Genes and genomic features are shown below. **B**) Top panel- schematic diagram of the *act1* gene with CDS sequence (in grey); the PCR amplicons used for the ChIP assay are indicated by the dotted lines above (numbers correspond to the primer positions relative to start codon). Bottom panel- polyacrylamide gels showing radiolabelled PCR products produced by the *act1* specific primer pairs (top bands) and by the pair specific for the intergenic region (bottom bands); using input DNA before ChIP (left panel) and using ChIP-enriched DNA from asynchronous (middle panel) and S-phase culture (right panel). The relative enrichment of *act1* DNA relative to intergenic sequence is expressed as a ratio of the intensity of the same fragments produced with the input DNA. **C**) Independent qPCR quantification of Upf1-Flag ChIP signal on 4 specific regions of *pma1* gene and 1 intergenic control in the absence and presence of RNase. N.T- not-tagged.

Figure S3

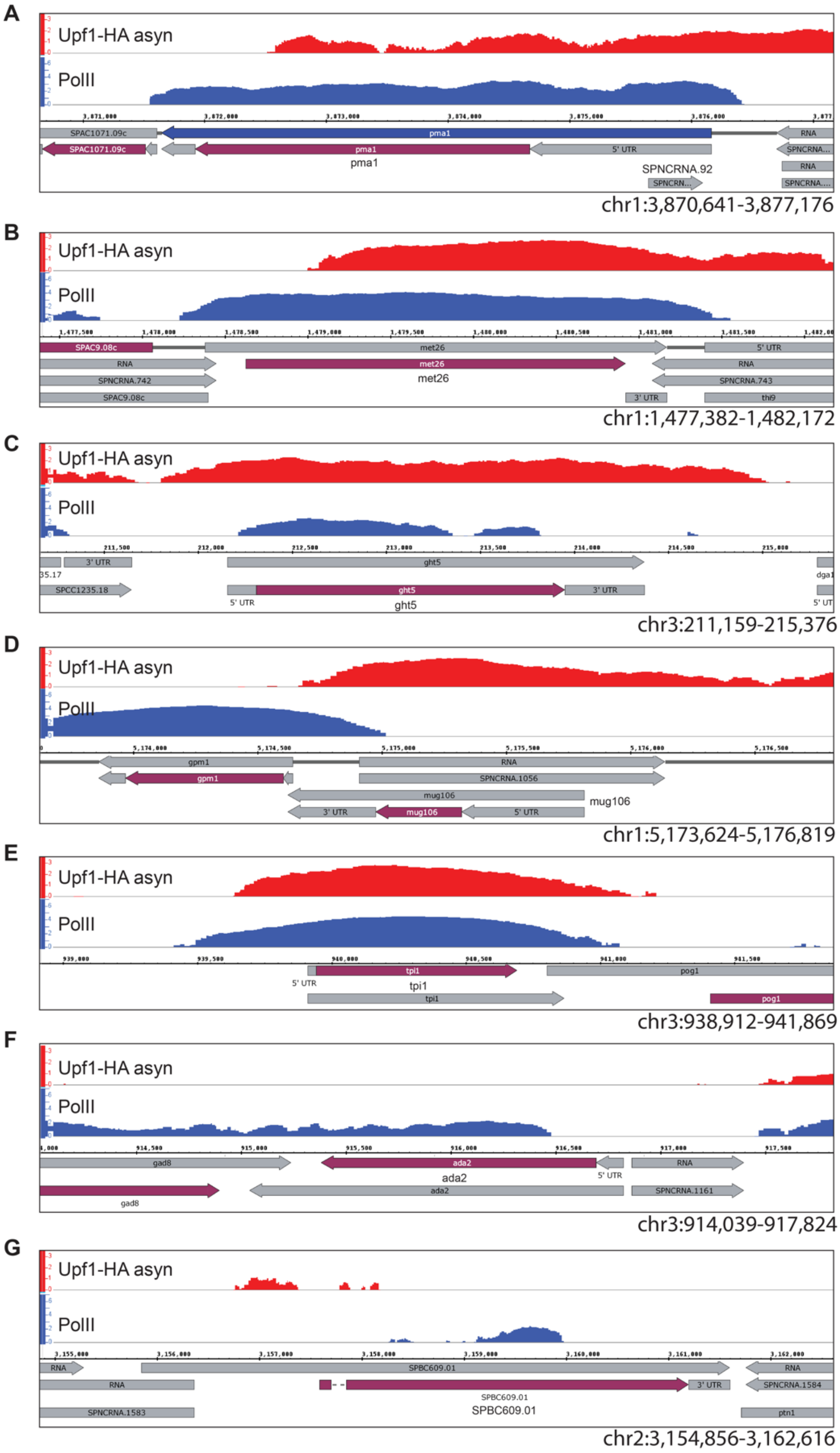
A
Upf1-HA asyn
PolII
pma1
SPAC1071.09c
5' UTR
SPNCRNA.92
RNA
chr1:3,870,641-3,877,176
B
Upf1-HA asyn
PolII
SPAC9.08c
met26
5' UTR
RNA
SPNCRNA.742
SPNCRNA.743
3' UTR
thi9
chr1:1,477,382-1,482,172
C
Upf1-HA asyn
PolII
3' UTR
ght5
SPCC1235.18
5' UTR
chr3:211,159-215,376
D
Upf1-HA asyn
PolII
gpm1
RNA
SPNCRNA.1056
mug106
3' UTR
5' UTR
chr1:5,173,624-5,176,819
E
Upf1-HA asyn
PolII
tpi1
5' UTR
pog1
chr3:938,912-941,869
F
Upf1-HA asyn
PolII
gad8
ada2
5' UTR
RNA
SPNCRNA.1161
chr3:914,039-917,824
G
Upf1-HA asyn
PolII
RNA
SPBC609.01
3' UTR
SPNCRNA.1584
SPNCRNA.1583
ptn1
chr2:3,154,856-3,162,616

Supplementary Figure S3. Upf1 and Ser5 Pol II ChIP-chip binding profiles at specific genes. IGB screenshot of ChIP-chip enrichments of Upf1-HA (top row) and Ser5 Pol II (bottom row) in asynchronous culture over seven different genes tested in this study. Genes and genomic features are shown below. The seven genes are **A**) *pma1*, **B**) *met26*, **C**) *ght5*, **D**) *mug-106*, **E**) *tpi1*, **F**) *ada2* and **G**) *SPBC609.01*. The coordinates of genes and flanking genomic regions are mentioned in the bottom right corners.

Figure S4

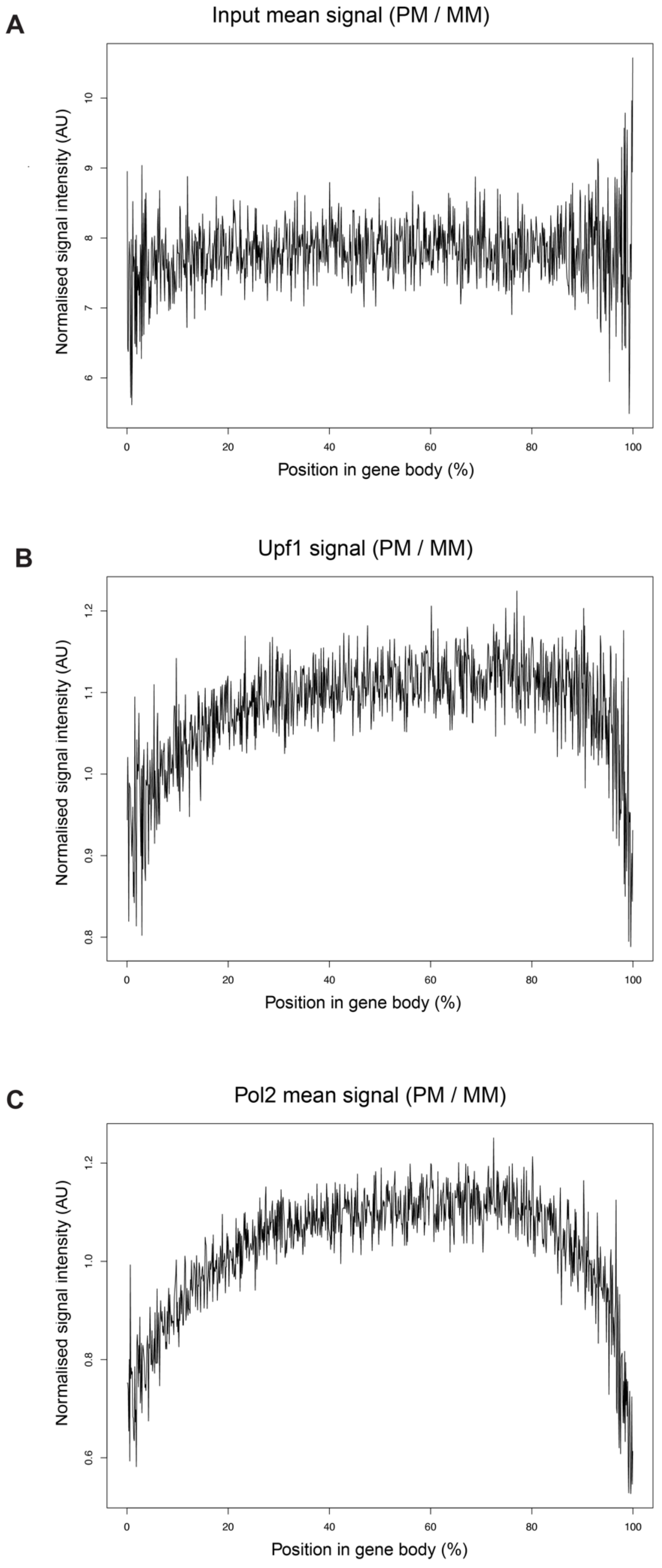
A
Input mean signal (PM / MM)
Normalised signal intensity (AU)
6
7
8
9
10
0
20
40
60
80
100
Position in gene body (%)
B
Upf1 signal (PM / MM)
Normalised signal intensity (AU)
0.8
0.9
1.0
1.1
1.2
0
20
40
60
80
100
Position in gene body (%)
C
Pol2 mean signal (PM / MM)
Normalised signal intensity (AU)
0.6
0.8
1.0
1.2
0
20
40
60
80
100
Position in gene body (%)

Supplementary Figure S4. Metagene profiles of Upf1 and Ser5 Pol II ChIP-chip enrichment data. **A**) Mean of the normalised signal intensity (PM (perfect match) / (mismatch) MM) for each probe in the two Upf1-related input control arrays, averaged for each position those probes map to through the gene body for all genes analysed. **B**) Normalised signal intensity (PM / MM) of the Upf1 IP array (S-phase), also normalised by dividing by the appropriate input control signal (shown in A), averaged for each position across all genes. **C**) Mean of the normalised signal intensity (PM / MM) for each probe in the two Ser5 Pol II IP arrays, also normalised by the corresponding input control signal (see Materials and Methods), and averaged for each position across all genes.

Figure S5

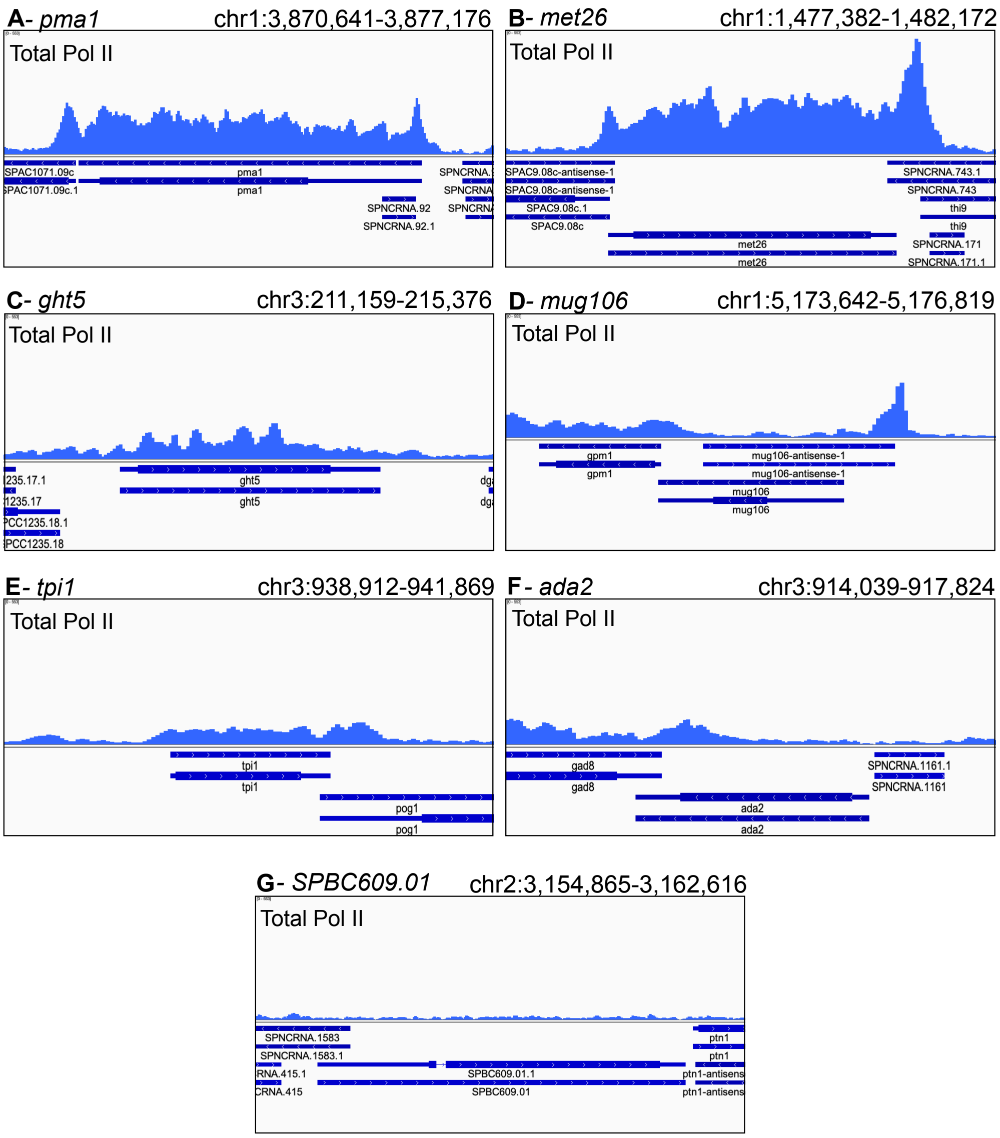

Supplementary Figure S5. Total Pol II loading profiles at selected genes. Integrated Genome Viewer screenshots of Rpb3 ChIP-seq enrichment profiles at the seven genes described in the study, and in Figure S3. The seven genes are **A**) *pma1*, **B**) *met26*, **C**) *ght5*, **D**) *mug*-106, **E**) *tpi1*, **F**) *ada2*, and **G**) SPBC609*.01*.

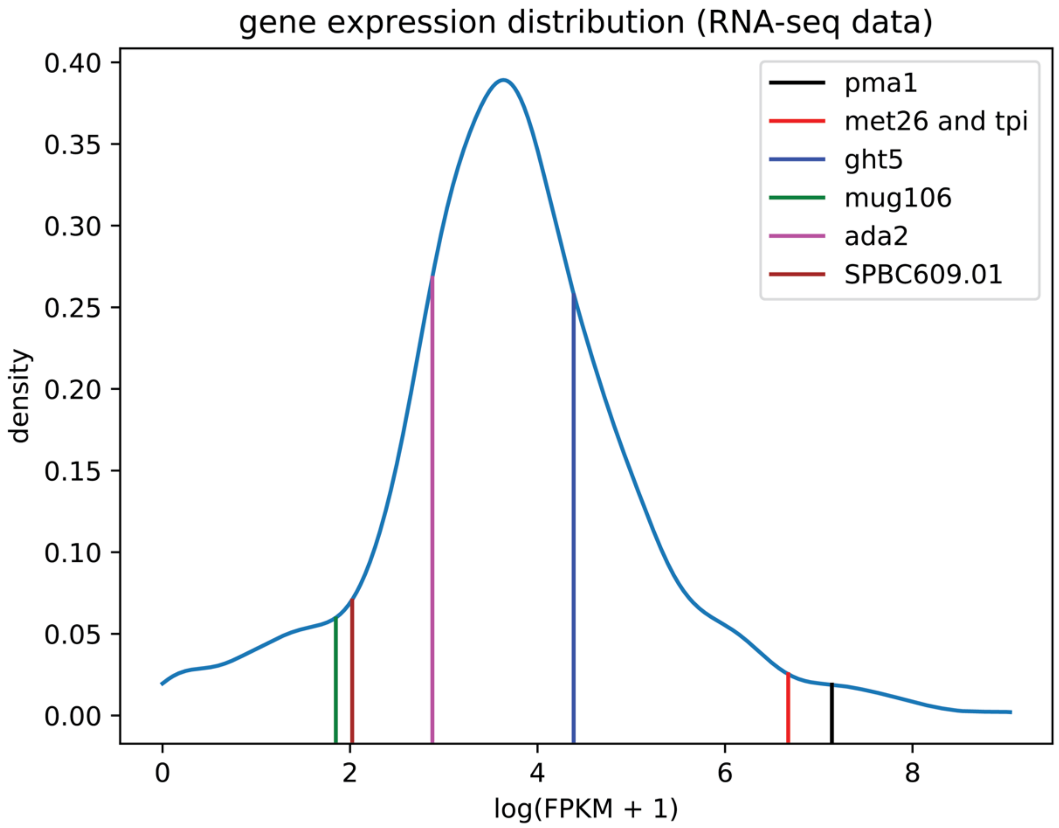

Supplementary Figure S6. Relative gene expression levels of selected genes. Density plot of the log-transformed FPKM+1 expression values of all active genes expressed in a wild-type *S. pombe*. Expression level of the seven genes discussed in the manuscript are indicated by vertical lines, which are shown in different colours, as labeled in the inset.

Figure S7

A

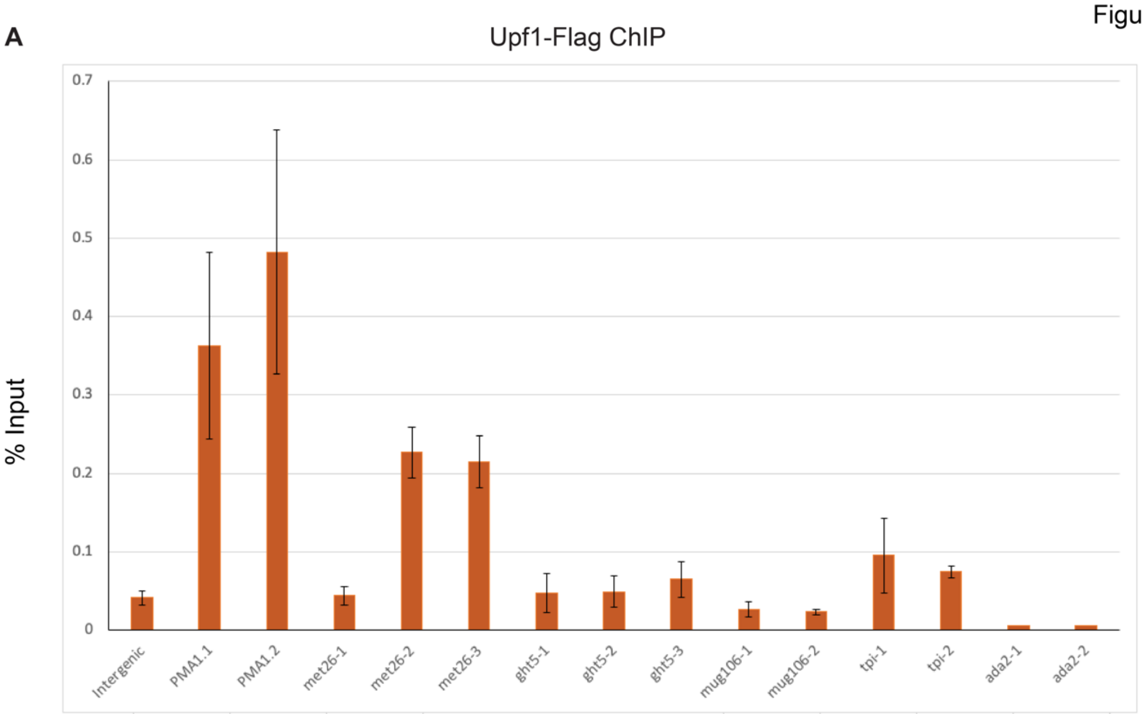
Upf1-Flag ChIP
% Input
0.7
0.6
0.5
0.4
0.3
0.2
0.1
0
Intergenic
PMA1.1
PMA1.2
met26-1
met26-2
met26-3
ght5-1
ght5-2
ght5-3
mug106-1
mug106-2
tpi-1
tpi-2
ada2-1
ada2-2

B

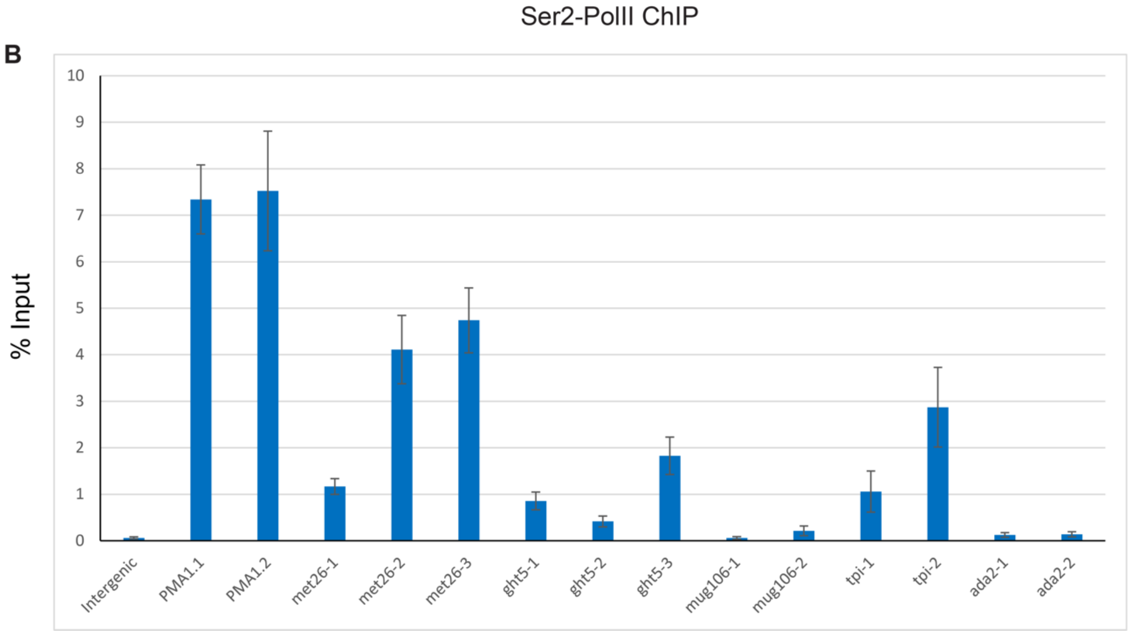
Ser2-PolII ChIP
% Input
10
9
8
7
6
5
4
3
2
1
0
Intergenic
PMA1.1
PMA1.2
met26-1
met26-2
met26-3
ght5-1
ght5-2
ght5-3
mug106-1
mug106-2
tpi-1
tpi-2
ada2-1
ada2-2

Supplementary Figure S7. Upf1 and Ser2 Pol II ChiP-qPCR enrichment of specific genes. ChIP-qPCR of biological replicates at the specific genomic regions depicted in Figure 2C and 2D. **A**) Upf1-Flag ChIP-qPCR on specific regions of six selected genes and one intergenic control (−1, −2 and −3 refer to separate amplicons of the named gene) **B**) Ser2 Pol II ChIP-qPCR of six selected genes and the intergenic control. Results are shown as percentage of input enrichment and correspond to the average of two biological replicates (mean ± SEM).

Figure S8

A

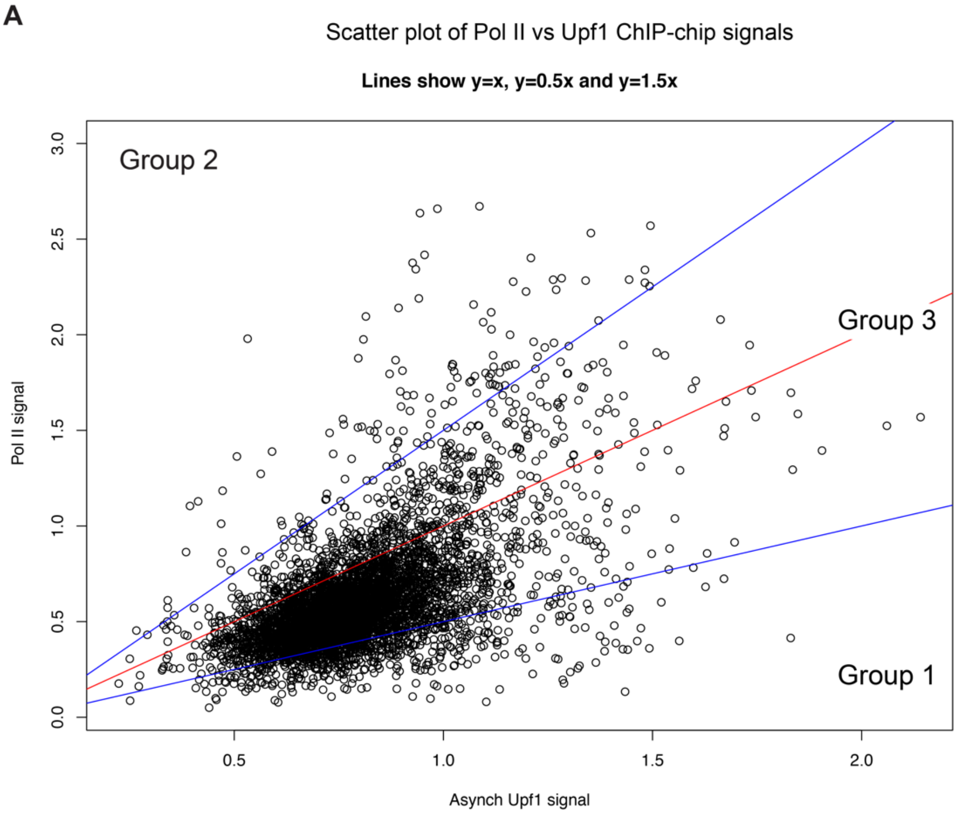
Scatter plot of Pol II vs Upf1 ChIP-chip signals
Lines show y=x, y=0.5x and y=1.5x
Group 2
Group 3
Group 1
Pol II signal
0.0
0.5
1.0
1.5
2.0
2.5
3.0
0.5
1.0
1.5
2.0
Asynch Upf1 signal

B

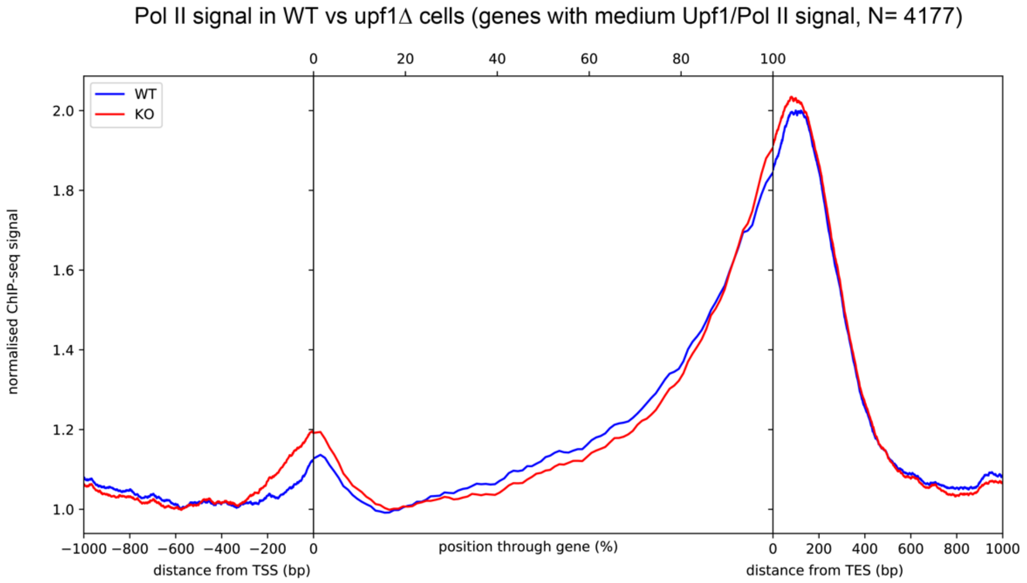
Pol II signal in WT vs upf1Δ cells (genes with medium Upf1/Pol II signal, N= 4177)
WT
KO
normalised ChIP-seq signal
1.0
1.2
1.4
1.6
1.8
2.0
0
20
40
60
80
100
−1000 −800 −600 −400 −200 0
distance from TSS (bp)
position through gene (%)
0 200 400 600 800 1000
distance from TES (bp)

Supplementary Figure S8. Genes grouping based on different Upf1 relative to Ser5 Pol II ChIP signals ratios. A) Scatter plot showing gene-specific ChIP-chip signal values for asynchronous Upf1 vs Ser5 Pol II IP arrays (see Methods and Materials). Pol II (y-axis) vs Upf1(x-axis) ChIP signals. Genes below the bottom blue line represent those having asynchronous Upf1 signal at least 50% higher than their Pol II signal (group 1, 587 genes). Genes above the top blue line represent those with asynchronous Upf1 signal at least 50% lower than their Pol II signal (group 2, 124 genes). Genes located between the two blue lines are classed as having medium Upf1 signal relative to Pol II (group 3, 4177 genes). The genes in these three groups are listed in Supplementary Table 4. **B**) Metagene analysis performed with genes from group 3. The averaged profiles for all the genes are displayed. The analysed regions cover the sequence 1000 bp upstream of the TSS, the gene body and 1000 bp downstream of the TES. The blue line shows wild-type (WT) and red line shows *Upf1*Δ profiles. The same metagene analyses were performed for group 1 and 2 and are shown in Figure 4B and 4C respectively.

Figure S9

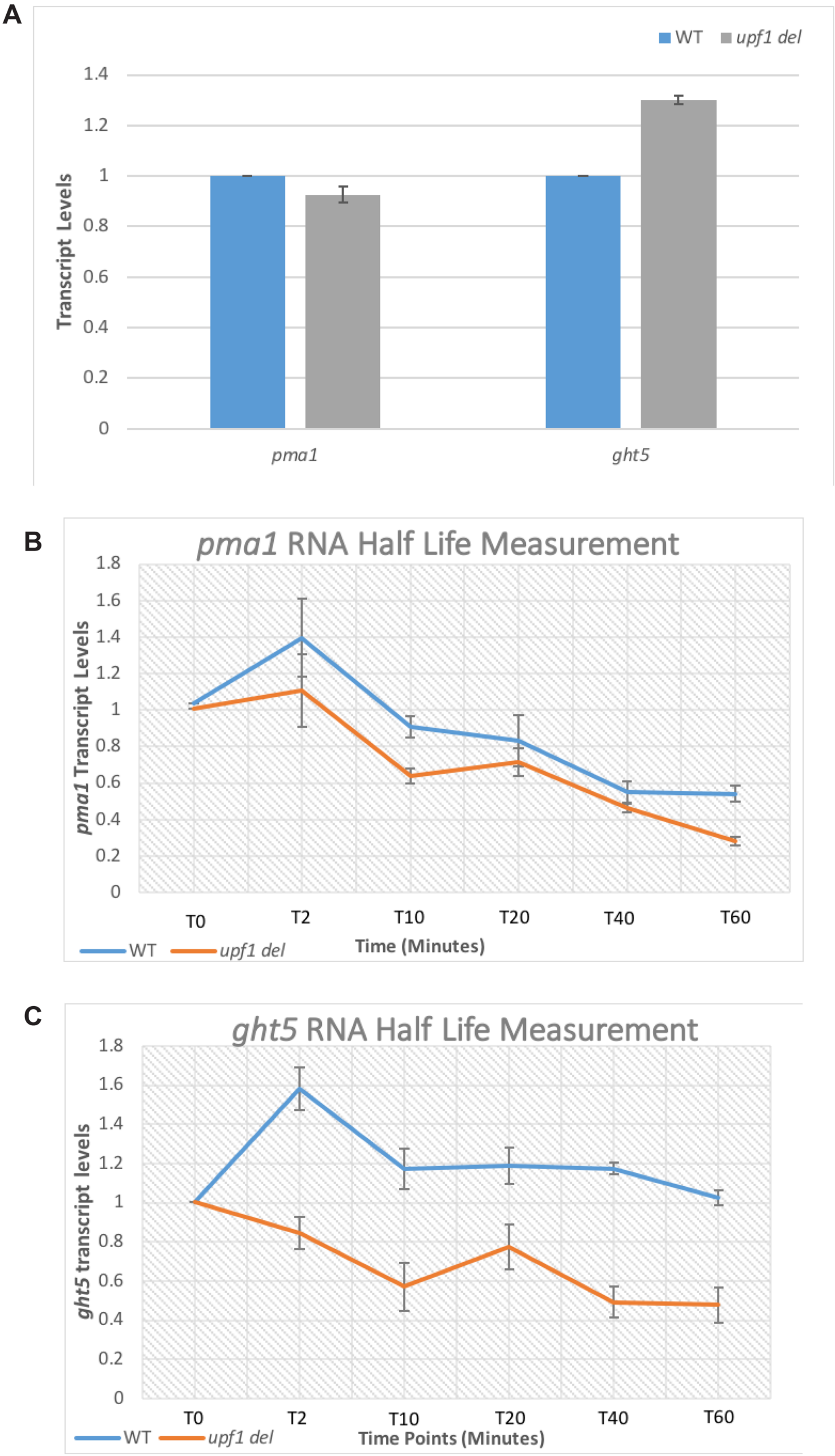
A
WT
upf1 del
1.4
1.2
1
0.8
0.6
0.4
0.2
0
Transcript Levels
pma1
ght5
B
pma1 RNA Half Life Measurement
1.8
1.6
1.4
1.2
1
0.8
0.6
0.4
0.2
0
pma1 Transcript Levels
T0
T2
T10
T20
T40
T60
Time (Minutes)
WT
upf1 del
C
ght5 RNA Half Life Measurement
1.8
1.6
1.4
1.2
1
0.8
0.6
0.4
0.2
0
ght5 transcript levels
T0
T2
T10
T20
T40
T60
Time Points (Minutes)
WT
upf1 del

Supplementary Figure S9. Decay profile of *pma1* and *ght5* in wild-type and *upf1*Δ.
A) qRT-PCR quantification of steady-state *ght5* and *pma1* transcript levels in WT and *upf1* deletion strains. Quantification is based on three biological replicates which are normalised using *RPL32* mRNA levels (mean ± SEM). **B**) Decay kinetics of *pma1*. qRT-PCR quantification of *pma1* mRNA levels at different time points after transcription inhibition with 1,10-phenanthroline (150µg/ml). Quantification corresponds to three biological replicates normalised by 18S rRNA levels (mean ± SEM). **C**) Similar decay kinetic experiment as described in B for *ght5* mRNA.

Figure S10

A

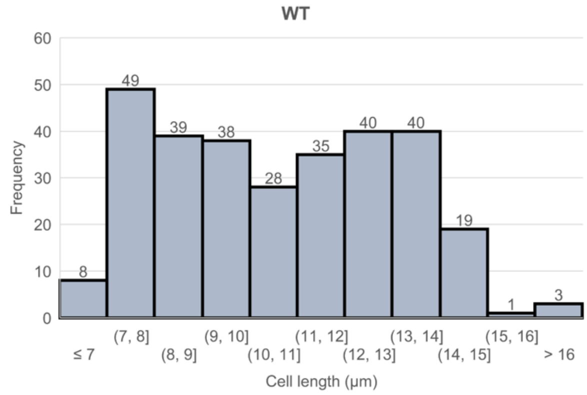

B

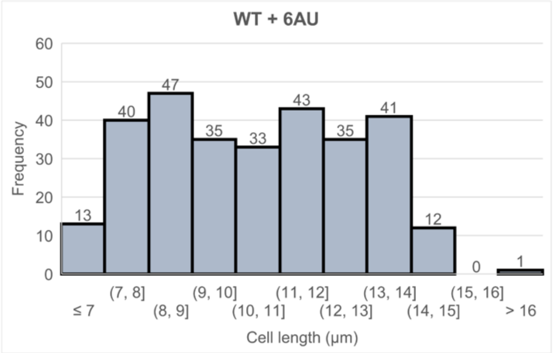

C

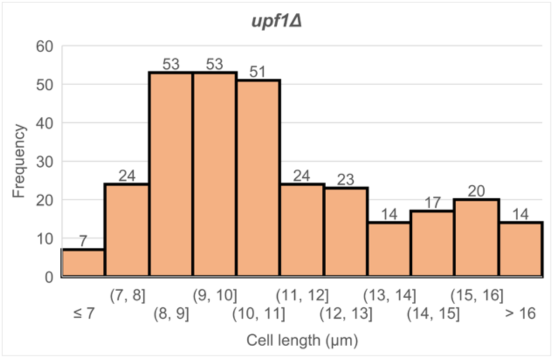

D

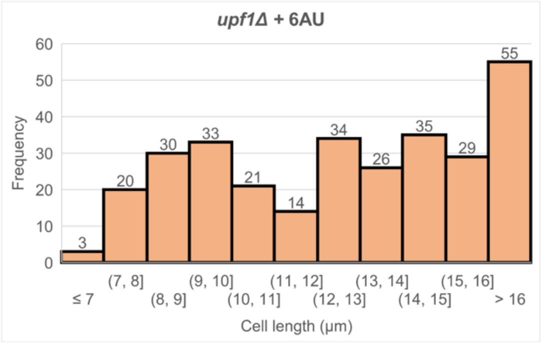

E

Condition	Wild-type	Wild-type + 6AU	*upf1Δ*
Wild-type + 6AU	1	—	—
upf1Δ	1	1	—
upf1Δ + 6AU	3.2×10^{-14}	2.8×10^{-16}	4.0×10^{-10}

Supplementary Figure S10. Cell length distribution in wild-type and *upf1*Δ cells treated with 6AU. Cell length distributions in **A**) wild-type, **B**) wild-type cells treated 6AU, **C**) *upf1*Δ and **D**) *upf1*Δ treated with 6AU. Quantification is based on 300 cells for each group. Phase contrast brightfield images of cells were acquired using a BH2 series microscope (Olympus) equipped with an external camera (GXCAM-Eclipse, GT Vision Ltd). Images were processed and the cell length measured using ImageJ. **E**) Kruskal-Wallis statistical tests were performed to compare the medians of all groups. Individual comparisons were performed with the pairwise-Wilcoxon test with Bonferroni adjustment, p-values for the six combinations are indicated. The median of *upf1*Δ 6AU treated cells is statistically significant to that of the cells belonging to all other experimental groups. This data was used to produce density plots and p-values shown in Figure 7B.

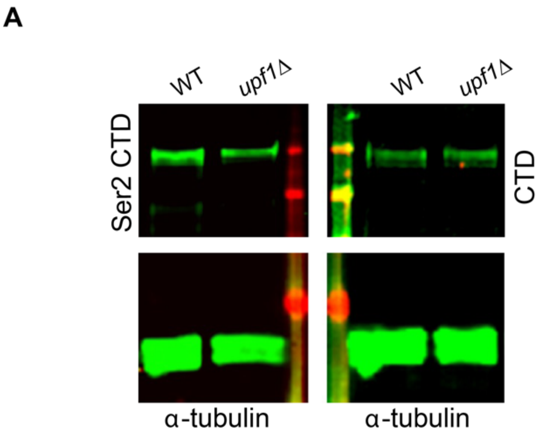

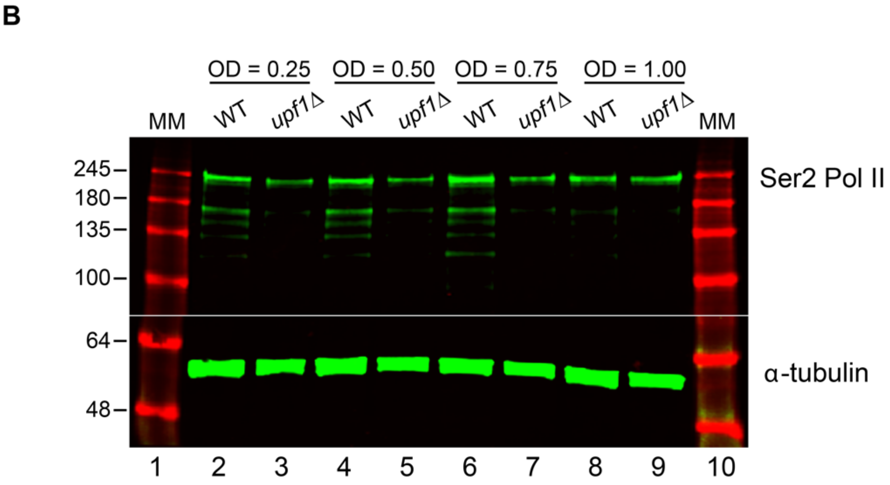

Supplementary Figure S11. Rbp1 displays slower gel mobility in *upf1*Δ

A) Western blotting of total S. pombe protein extract from cells at OD600 0.5, blot on the left incubated with Ser2 CTD antibody, blot on the right incubated with unphosphorylated CTD antibody. Bottom panels were incubated with alpha-tubulin antibody as loading control. B) Western blotting of different time points from a growing culture (the OD600 of the culture at each point is indicated above). The top half of the membrane was incubated with a Ser2 Pol II antibody and the bottom half with an alpha-tubulin antibody as in A. Lanes 1 and 10 show the same protein molecular weights marker (values are in kilodaltons).

Supplementary data files description

The two supplementary html files show plots of the normalised single-base resolution coverage (Materials and Methods) of the Pol II ChIP-seq signal for two different sets of genes. Two plots are shown for each gene. The first plot compares the coverage depth from 1000bp upstream of the TSS to 1000bp downstream of the TES in wild-type (WT, blue line) and *upf1*Δ (termed KO, red line) Upf1 cells for that particular gene. This is referred to as the "normalised ChIP-seq signal" in the y-axis. The second plots show the difference between the WT and *upf1*Δ signals, so that more negative values indicate increased Pol II occupancy when Upf1 is deleted. The vertical black line on the left side of each plot marks the TSS and the vertical black line on the right marks the position of the TES. The gene that each plot corresponds to is indicated by the ID in the plot title.

Supplementary Data File 1

The genes plotted in supplementary file 1 are a selection of those found to be strongly associated with Upf1 in WT cells, which also exhibit increased Pol II occupancy throughout the gene body, at the TES and/or downstream of the TES in *upf1*Δ (termed KO) cells, as demonstrated by the plots. This selection does not include the genes that are misregulated in *upf1*Δ cells, which are instead shown separately in Supplementary File 2 below.

Supplementary Data File 2

The genes plotted in Supplementary File 2 are a selection of those found to be both strongly associated with Upf1 in WT cells and misregulated in *upf1*Δ (termed KO) cells, which also exhibit increased Pol II occupancy throughout the gene body, at the TES and/or downstream of the TES in *upf1*Δ cells.
